# CRISPR screening identifies the deubiquitylase ATXN3 as a PD-L1–positive regulator for tumor immune evasion

**DOI:** 10.1172/JCI167728

**Published:** 2023-12-01

**Authors:** Shengnan Wang, Radhika Iyer, Xiaohua Han, Juncheng Wei, Na Li, Yang Cheng, Yuanzhang Zhou, Qiong Gao, Lingqiang Zhang, Ming Yan, Zhaolin Sun, Deyu Fang

**Affiliations:** 1 Department of Pathology, Northwestern University Feinberg School of Medicine, Chicago, Illinois, USA.; 2 College of Basic Medical Sciences, Dalian Medical University, Dalian, China.; 3 Department of Physiology, School of Basic Medicine, Qingdao University, Qingdao, Shandong, China.; 4 State Key Laboratory of Proteomics, National Center of Protein Sciences (Beijing), Beijing Institute of Lifeomics, Beijing, China.; 5 Department of Oral Maxillofacial Head and Neck Oncology, Shanghai Ninth People’s Hospital, College of Stomatology, Shanghai Jiao Tong University School of Medicine, National Clinical Research Center for Oral Diseases, Shanghai Key Laboratory of Stomatology and Shanghai Research Institute of Stomatology, Shanghai, China.

**Keywords:** Otology, Immunotherapy, Lung cancer, Ubiquitin-proteosome system

## Abstract

Regulation of tumoral PD-L1 expression is critical to advancing our understanding of tumor immune evasion and the improvement of existing antitumor immunotherapies. Herein, we describe a CRISPR-based screening platform and identified ATXN3 as a positive regulator for PD-L1 transcription. TCGA database analysis revealed a positive correlation between *ATXN3* and *CD274* in more than 80% of human cancers. ATXN3-induced *Pd-l1* transcription was promoted by tumor microenvironmental factors, including the inflammatory cytokine IFN-γ and hypoxia, through protection of their downstream transcription factors IRF1, STAT3, and HIF-2α. Moreover, ATXN3 functioned as a deubiquitinase of the AP-1 transcription factor JunB, indicating that ATNX3 promotes PD-L1 expression through multiple pathways. Targeted deletion of ATXN3 in cancer cells largely abolished IFN-γ– and hypoxia-induced PD-L1 expression and consequently enhanced antitumor immunity in mice, and these effects were partially reversed by PD-L1 reconstitution. Furthermore, tumoral ATXN3 suppression improved the preclinical efficacy of checkpoint blockade antitumor immunotherapy. Importantly, ATXN3 expression was increased in human lung adenocarcinoma and melanoma, and its levels were positively correlated with PD-L1 as well as its transcription factors IRF1 and HIF-2α. Collectively, our study identifies what we believe to be a previously unknown deubiquitinase, ATXN3, as a positive regulator for *PD-L1* transcription and provides a rationale for targeting ATXN3 to sensitize checkpoint blockade antitumor immunotherapy.

## Graphical abstract

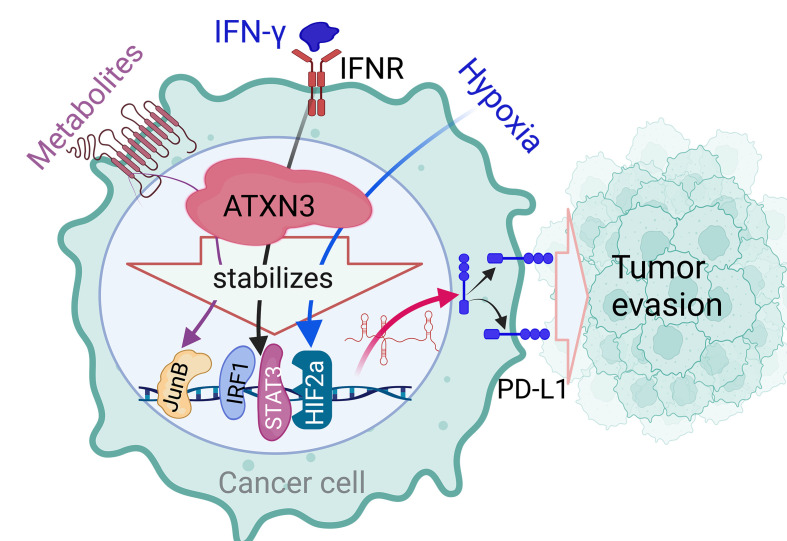

## Introduction

Antitumor immune therapy, particularly through the release of negative regulators of immune activation or immune checkpoints that limit antitumor responses, has achieved tremendous success in the treatment of a variety of cancers over the last decade (1, 2). This can be achieved by antibodies that block the cytotoxic T lymphocyte–associated protein 4 (CTLA-4) or the programmed cell death 1 (PD-1) pathway, either alone or in combination. Tumor cells often express PD-1 ligand-1 (PD-L1), also known as B7 homolog 1 (B7-H1) or CD274, a type 1 transmembrane protein that leads to the inhibition of PD-1–positive T lymphocyte proliferation, cytokine production, and cytolytic activity (3). PD-L1 expression is regulated by intrinsic oncogenic and adaptive signaling pathways to facilitate cancer immunosurveillance escape in the tumor microenvironment at transcriptional and posttranslational levels. For instance, the oncogenic transcription factor MYC, which is often upregulated in a variety of types of cancer, can directly bind to the PD-L1 promoter, and enhance its expression (4). Another driver of PD-L1 upregulation is hyperactivation of anaplastic lymphoma kinase (ALK), caused by nucleophosmin-ALK gene fusion, which promotes PD-L1 expression via STAT3 transcription factor (5). In addition, tumoral PD-L1 expression is regulated by tumor microenvironmental factors such as hypoxia, metabolites, and cytokines (6). Under hypoxia conditions, both HIF-1α and HIF-2α transcription factors can directly promote *CD274* gene transcription (6–8). Moreover, cytokines produced by tumor cells and/or infiltrated immune cells, such as TGF-β, TNF-α, and IFN-γ, induce *CD274* gene transcription (9, 10).

In addition, post-transcriptional modifications including phosphorylation, ubiquitination, acetylation, and glycosylation are involved in regulating the protein stability of PD-L1 (11). Glycosylation is known to stabilize PD-L1, and although fully glycosylated PD-L1 has a half-life of more than 12 hours, non-glycosylated PD-L1 undergoes rapid proteolysis, with a half-life of less than 4 hours (11). Several ubiquitin modification enzymes that control PD-L1 ubiquitination-induced degradation have been identified. The SCFβ-TrCP E3 ubiquitin ligase is known for degrading non-glycosylated PD-L1 through K48-linked polyubiquitination (12). In contrast, HMG-CoA reductase degradation protein 1 (HRD1), an E3 ligase involved in the endoplasmic reticulum stress response and in regulating both T and B cell immunity (13–16), targets PD-L1 with abnormal glycosylation for ubiquitination-mediated degradation (17). PD-L1 is also a substrate of STIP1 homology and U-box–containing protein 1 (STUB1) in polyubiquitination and downregulation of membrane-bound of PD-L1 (18). In addition, speckle-type POZ protein (SPOP), an E3 ubiquitin ligase adaptor protein, stabilizes PD-L1 through cyclin D/cyclin-dependent kinase 4 in late G_1_ and S phases (19). On the other hand, several deubiquitinating enzymes, including ubiquitin-specific peptidase 7 (USP7), USP22, OTUB1, and CSN5, stabilize PD-L1 protein in cancer cells (20–23). However, the involvement of deubiquitinases in regulating *CD274* gene transcription remains unidentified.

Herein, we used an unbiased CRISPR screening approach for all deubiquitinase family members and identified ATXN3 as the top positive regulator of PD-L1 transcription. ATXN3 functions as a deubiquitinase for multiple transcription factors of PD-L1 in tumor cells in response to tumor microenvironmental factors. Targeted deletion of ATXN3 resulted in a dramatic reduction in PD-L1 transcription, which consequently improved antitumoral immunity by synergizing with checkpoint blockade therapy. Our study identifies ATXN3 as a positive regulator of *CD274* gene transcription and a potential therapeutic target to enhance antitumor immune therapy.

## Results

### Identification of ATXN3 as a PD-L1–positive regulator through unbiased CRISPR screening.

**Figure 1:**
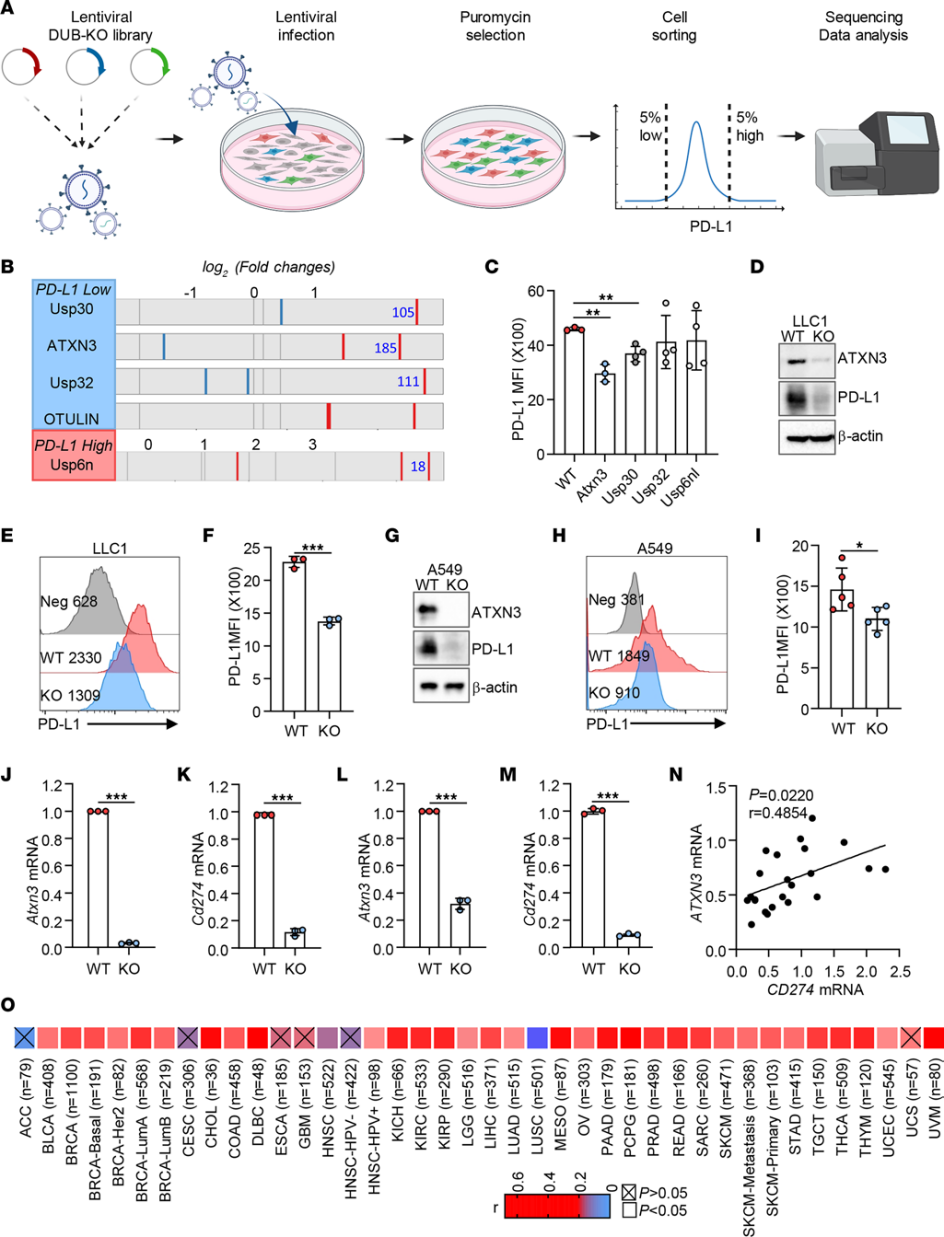
Identification of ATXN3 as a PD-L1–positive regulator in cancer cells by unbiased CRISPR screening. (**A**) Schematic of the deubiquitinase CRISPR knockout screening workflow. (**B**) Guides enriched in PD-L1–low and PD-L1–high populations with their fold enrichment. The guide code of each gene for further validation is indicated. (**C**) Guide hits described were validated by flow using individual guide knockouts. (**D**) Western blotting validation of ATXN3 knockout and PD-L1 expression with specific sgRNAs in LLC1 cells. WT, cells transfected with empty vector; KO, ATXN3-knockout stable cell strains. (**E** and **F**) Representative flow cytometry plots and quantification by MFI of cell-surface PD-L1 in LLC1 cells. (**G**) Western blotting analysis of ATXN3 and PD-L1 expression in A549 cells with knockout of *ATXN3*. (**H** and **I**) Representative flow cytometry plots and quantification of cell-surface PD-L1 in A549 cells with knockout of *ATXN3*. (**J** and **K**) *Cd274* and *Atxn3* mRNA levels were analyzed by reverse transcription quantitative PCR (RT-qPCR) in LLC1 cells. (**L** and **M**) *Cd274* and *Atxn3* mRNA levels were analyzed by RT-qPCR in B16 cells. (**N**) Correlation of *CD274* mRNA levels with *ATXN3* mRNA levels in lung cancer patients (*n =* 22). (**O**) Correlation of *CD274* with *ATXN3* expression in multiple tumors based on TCGA data (*n =* 40). **C**, **F**, and **I**–**M**: 2-tailed unpaired *t* test; **N**: Pearson’s correlation analysis. *P < 0.05, **P < 0.01, ***P < 0.001.

To identify the specific deubiquitinases that promote tumoral PD-L1 expression, we first designed a targeted library of all 96 mammalian deubiquitinase family members based on the optimized single-guide RNA (sgRNA) sequences and cloned each of them into a lentivirus-based lentiCRISPR v2 vector system, which coexpresses CRISPR-associated endonuclease 9 (Cas9) (Supplemental Figure 1A; supplemental material available online with this article; https://doi.org/10.1172/JCI167728DS1). The pooled CRISPR plasmids were further validated by sequencing for their equal representation and then transfected into Lenti-X packaging cells. The pooled viruses were titrated as reported (24); B16 melanoma cells, which express a high level of PD-L1, were infected with 0.3 multiplicity of infection of the pooled DUB-KO lentivirus library; and, 48 hours after transduction, cells were selected with 2 μg/mL of puromycin for 3–4 days. The infectivity of selected cancer cells was further validated by intracellular staining of Cas9 (Supplemental Figure 1, B and C). The puromycin-selected cells were then sorted for PD-L1–low and –high populations to identify positive and negative PD-L1 regulators, respectively (Supplemental Figure 1D). Genomic DNA was purified from the sorted cells, and the specific region carrying the guide sequences was amplified by a 1-step PCR for sequencing (Figure 1A). Guides enriched in either PD-L1–low or –high populations were analyzed using MAGeCK count and test functions. Genes were ranked using the MAGeCK radioreceptor assay enrichment score. Fold change in read counts of each sgRNA within the top genes was analyzed. Guides were selected for further validation based on *P* values (Supplemental Figure 1, E–H). A total of 4 deubiquitinases, ATXN3, USP30, USP32, and OTULIN, were enriched in PD-L1–low populations, which are potential PD-L1–positive regulators (Supplemental Figure 1, E and F, and Figure 1B). USP30 was recently shown to promote tumoral PD-L1 expression (25), providing confidence for our screening. On the other hand, while 4 deubiquitinases, USP6NL, USP18, USP27X, and OTUD3, were exclusively enriched in the PD-L1–high pool of B16 cells, only USP6NL reached statistical significance, implicating USP6NL as a negative PD-L1 regulator (Supplemental Figure 1, G and H, and Figure 1B). Importantly, further analysis of CRISPR KO B16 melanoma cells confirmed that targeted suppression of ATXN3 and USP30 dramatically reduced PD-L1 expression (Figure 1C). It has been reported that both USP7 and USP22 protect PD-L1 from ubiquitination-mediated degradation in cancers including gastric cancer, lung adenocarcinoma, and liver cancer (20-23), neither of which was enriched in our screening (Figure 1B). This is likely explained by regulation of PD-L1 by USP7 and USP22 in a tumor type–specific manner, since we have shown previously that CRISPR targeted USP22 deletion resulted in PD-L1 downregulation in human breast cancer cells but not in B16 cells (26). Nevertheless, our CRISPR screening identified ATXN3 as a previously unknown positive regulator in cancer cells.

### ATXN3 promotes PD-L1 expression at transcriptional level in a broad spectrum of cancer cells.

We then validated the potential role of ATXN3 in regulating tumoral PD-L1 expression in mouse and human lung cancer cells. CRISPR deletion of ATXN3 expression in Lewis lung carcinoma LLC1 cells resulted in a significant reduction in PD-L1 protein expression as detected by Western blotting and flow cytometry (Figure 1, D–F). Furthermore, CRISPR-mediated ATXN3 deletion dramatically reduced PD-L1 expression in mouse B16 melanoma, colon cancer MC38, and triple-negative breast cancer 4T1 cells (Supplemental Figure 2) and human lung small cell adenocarcinoma A549 cells (Figure 1, G–I), which was further confirmed by an alternative approach using shRNA-mediated knockdown of the ATXN3 gene (Supplemental Figure 3, A and B). In contrast, only a modest but statistically significant reduction in PD-L1 expression was detected in human colon cancer HCT116 cells by ATXN3-targeted knockdown (Supplemental Figure 3, C and D). This is likely due to HCT116 expressing low levels of ATXN3 endogenously (Supplemental Figure 3E). Interestingly, ATXN3 appears to positively regulate PD-L1 expression at the transcriptional level, since real-time reverse transcription PCR analysis confirmed that *Atxn3* targeting dramatically inhibited *Cd274* mRNA expression in both LLC1 and B16 mouse cancer cells (Figure 1, J–M). To support this conclusion, we further demonstrated that ATXN3 expression dramatically enhanced luciferase reporter activity under the control of an optimal 2 kb human *CD274* promoter region (Supplemental Figure 4). These results indicate that ATXN3 is a positive regulator of PD-L1 gene expression in a variety of both human and mouse cancer cells at the transcriptional level.

To further validate our findings in samples from patients with cancer, we first assessed mRNA expression levels of *ATXN3* and *CD274* in 22 lung cancer patients and found a positive correlation between *ATXN3* and *CD274* expression (Figure 1N). Consistently, analysis of the The Cancer Genome Atlas (TCGA) database revealed a positive correlation between *ATXN3* and *CD274* expression in human lung adenocarcinoma (Figure 1O). Importantly, in addition to lung adenocarcinoma, the positive correlation between *ATXN3* and *CD274* expression was identified in 33 of 40 total types of human cancers, including bladder urothelial carcinoma, breast invasive carcinoma, cholangial carcinoma, colon adenocarcinoma, lymphoid neoplasm diffuse large B cell lymphoma, head and neck squamous cell carcinoma–HPV^+^, kidney chromophobe, kidney renal clear cell carcinoma, kidney renal papillary cell carcinoma, brain lower-grade glioma, liver hepatocellular carcinoma, mesothelioma, ovarian serous cystadenocarcinoma, pancreatic adenocarcinoma, pheochromocytoma and paraganglioma, prostate adenocarcinoma, rectal adenocarcinoma, sarcoma, skin cutaneous melanoma, stomach adenocarcinoma, testicular germ cell tumors, thyroid carcinoma, thymoma, uterine corpus endometrial carcinoma, and uveal melanoma (Figure 1O), indicating that ATXN3 is a potential positive regulator of PD-L1 transcription in a broad spectrum of human cancers.

### ATXN3 is a positive regulator of IFN-γ–induced PD-L1 transcription in tumor cells.

**Figure 2:**
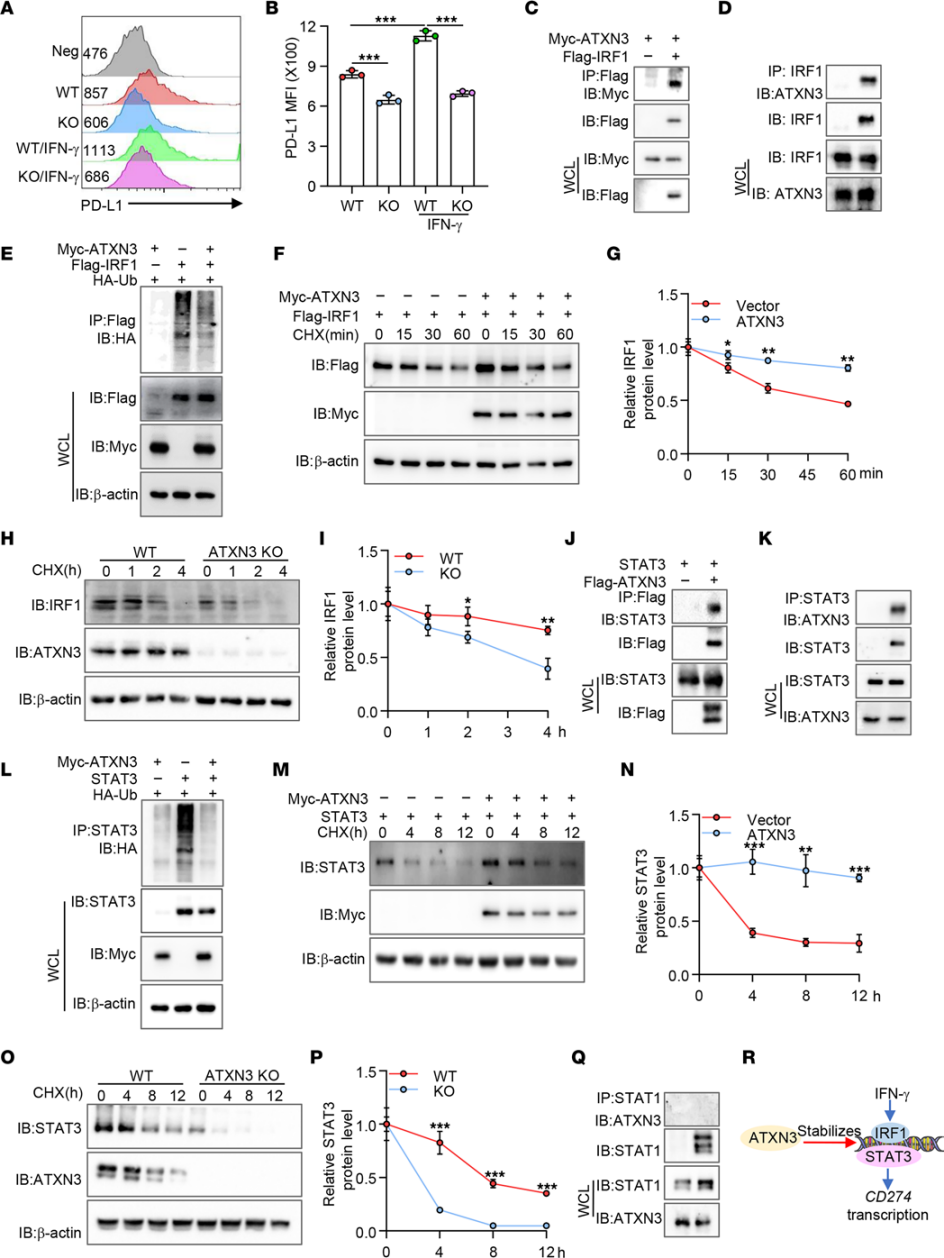
ATXN3 potentiates IFN-γ–induced PD-L1 expression through stabilizing IRF1 and STAT3. (**A** and **B**) WT and ATXN3-KO cells were treated with IFN-γ (10 ng/mL) for 24 hours, and surface PD-L1 levels were analyzed. (**C**) ATXN3 interacts with IRF1 in transiently transfected HEK293T cells. (**D**) Interaction of endogenous ATXN3 and IRF1 in A549 cells. (**E**) HA-ubiquitin and FLAG-IRF1 expression plasmids were cotransfected with Myc-ATXN3 into HEK293T cells. IRF1 ubiquitination was determined by immunoprecipitation of IRF1 and immunoblotting with HA antibody. (**F** and **G**) FLAG-IRF1 was cotransfected with or without Myc-ATXN3 plasmids into HEK293T cells. The transfected cells were treated with cycloheximide (CHX) for different times. The protein levels of FLAG-IRF1 (top panel) and Myc-ATXN3 (middle panel) with β-actin control (bottom panel) were analyzed by Western blotting. Representative images (**F**) and quantification data from 3 independent experiments are shown (**G**). (**H** and **I**) Immunoblot analysis of IRF protein stability in WT and ATXN3-KO A549 cells as in **F** and **G**. (**J**) Interaction between ATXN3 and STAT3 in transfected HEK293T cells. (**K**) Endogenous interaction between ATXN3 and STAT3 in A549 cells. (**L**) The effect of ATXN3 on STAT3 ubiquitination was determined as in **E**. (**M** and **N**) The effects of ATXN3 on STAT3 protein stability were analyzed as in **F** and **G**. (**O** and **P**) Immunoblot analysis of STAT3 protein stability in WT and ATXN3-KO A549 cells as in **H** and **I**. (**Q**) The interaction between ATXN3 and STAT1 was tested in A549 cells. (**R**) ATXN3 enhances tumoral PD-L1 expression through protecting IRF1 and STAT3 from ubiquitination-induced protein degradation. **B**: Ordinary 1-way ANOVA; **G**, **I**, **N**, and **P**: 2-tailed unpaired *t* test; *P < 0.05, **P < 0.01, ***P < 0.001. WCL, whole-cell lysate.

Tumoral PD-L1 expression is regulated by intrinsic oncogenic and adaptive signaling pathways. We first analyzed the possible involvement of ATXN3 in regulating PD-L1 expression induced by tumor microenvironmental cytokines, such as IFN-γ (27–29). As expected, treatment of human lung cancer A549 cells with IFN-γ resulted in a significant increase in PD-L1 expression. Targeted deletion of *ATXN3* by CRISPR largely abrogated IFN-γ–induced PD-L1 expression (Figure 2, A and B). Downstream transcription factors such as IRF1 are involved in IFN-γ–induced PD-L1 expression (10, 28). Indeed, coimmunoprecipitation (co-IP) and Western blotting detected that ATXN3 interacts with IRF1 in transiently transfected HEK293T cells (Figure 2C). The endogenous interaction between IRF1 and ATXN3 was further confirmed in human lung cancer A549 cells (Figure 2D), indicating that ATXN3 may enhance IFN-γ–induced PD-L1 expression through IRF1.

To determine the functional consequence of the interaction between ATXN3 and IRF1, we analyzed the effect of gain of the deubiquitinase ATXN3 functions on IRF1 ubiquitination. The gradual ubiquitination of IRF1 was detected in anti–FLAG-IRF1 immunoprecipitants when IRF1 and HA-ubiquitin were both expressed. Further expression of ATXN3 dramatically inhibited IRF1 ubiquitination (Figure 2E), indicating that ATXN3 is a deubiquitinase of IRF1. Consequently, gain of ATXN3 expression increased expression of IRF1 and its protein stability (Figure 2, F and G). Conversely, targeted ATXN3 deletion facilitated IRF1 protein degradation (Figure 2, H and I). Collectively, these observations indicate that ATXN3 promotes IFN-γ–induced PD-L1 transcription at least partially through the stabilization of its transcriptional factor IRF1 in cancer cells.

In addition to IRF1, the transcription factor STAT3 has been identified as a downstream transcription factor for PD-L1 expression (5, 30), raising a possibility that ATXN3 may also promote PD-L1 expression through STAT3. In fact, STAT3 interaction with ATXN3 was detected in transiently transfected HEK293T cells as well as endogenously in human lung cancer A549 cells (Figure 2, J and K). Expression of ATXN3 largely diminished STAT3 ubiquitination (Figure 2L). Therefore, gain of ATXN3 function increased STAT3 protein expression and prolonged its half-life (Figure 2, M and N). Conversely, loss of ATXN3 resulted in reduced STAT3 expression due to the elevated protein degradation (Figure 2, O and P). In contrast, ATXN3 interaction with another member of the STAT family, STAT1, which promotes PD-L1 transcription (10, 31), was undetected in A549 cells (Figure 2Q). Notably, ectotrophic expression of both IRF1 and STAT3 largely restored IFN-γ–induced PD-L1 expression in ATXN3-null lung cancer cells. In contrast, coexpression of both IRF1 and STAT3 failed to rescue PD-L1 expression when cells were cultivated under hypoxic conditions (Supplemental Figure 5A). Therefore, our data indicate that ATXN3 enhances IFN-γ–induced tumoral PD-L1 expression by protecting IRF1 and STAT3 from ubiquitination-induced protein degradation (Figure 2R).

### ATXN3 is a positive regulator for tumoral PD-L1 transcription under hypoxia conditions.

**Figure 3:**
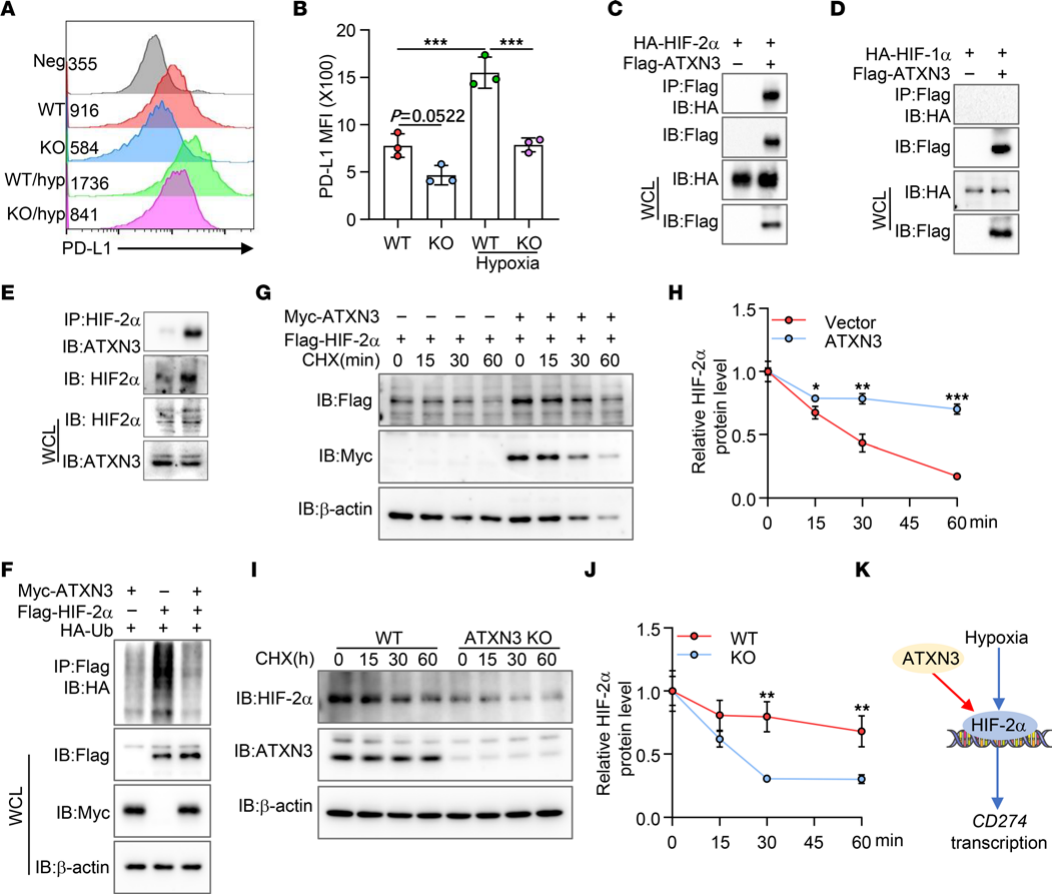
ATXN3 selectively functions as a HIF-2α deubiquitinase to promote tumoral PD-L1 transcription. (**A** and **B**) A549 cells were cultured under normoxia and hypoxia (hyp) (1% pO_2_) for 48 hours, and surface PD-L1 levels were analyzed by flow cytometry and quantification. (**C**) ATXN3 specifically interacts with HIF-2α. HA–HIF-2α expression plasmid was cotransfected with or without FLAG-ATXN3 into HEK293T cells. Their interactions were examined by co-IP with anti-FLAG antibodies and by Western blotting with anti-HA antibodies. (**D**) The interaction between ATXN3 and HIF-1α was tested in transfected HEK293T cells. (**E**) Endogenous interaction between ATXN3 and HIF-2α in A549 cells. (**F**) HA-ubiquitin, FLAG–HIF-2α, and Myc-ATXN3 plasmids were cotransfected into HEK293T cells. HIF-2α ubiquitination was determined by immunoprecipitation of HIF-2α with anti-FLAG antibodies and immunoblotting with anti-HA antibody. (**G** and **H**) HIF-2α was cotransfected with or without ATXN3 plasmids into HEK293T cells. The transfected cells were treated with CHX for different times. The protein levels of HIF-2α (top panel) and ATXN3 (middle panel) were analyzed by Western blotting. β-Actin was used as a loading control (bottom panel). (**I** and **J**) Immunoblot analysis of HIF-2α protein stability in WT and ATXN3-KO A549 cells. (**K**) ATXN3 enhances hypoxia-induced PD-L1 expression through protecting HIF-2α from ubiquitination-induced protein degradation. **B**: Ordinary 1-way ANOVA; **H** and **J**: 2-tailed unpaired *t* test. *P < 0.05, **P < 0.01, ***P < 0.001.

In addition to IFN-γ, tumor microenvironmental factors, such as hypoxia, have been known to strongly upregulate PD-L1 expression (6–8). Consistent with previous reports, PD-L1 expression on the surface of A549 cells was significantly increased under hypoxic conditions. Importantly, this hypoxia-induced PD-L1 upregulation was largely, while not totally, diminished by ATXN3 targeted deletion (Figure 3, A and B), implying a possibility that ATXN3 enhances hypoxia-induced PD-L1 expression in tumor cells. Since both HIF-1α and HIF-2α have been identified as PD-L1 transcription factors in cancer cells in response to hypoxia (6–8), we then reasoned whether ATXN3 enhances tumoral PD-L1 transcription through stabilizing HIF family transcription factors. Indeed, co-IP and Western blotting detected ATXN3 interaction with HIF-2α, but not HIF-1α, in transiently transfected HEK293T cells (Figure 3, C and D), implying a possibility that ATXN3 enhances hypoxia-induced PD-L1 expression specifically through HIF-2α. To support this notion, we confirmed endogenous interaction of ATXN3 with HIF-2α in human lung cancer A549 cells (Figure 3E).

A deubiquitinase often inhibits the ubiquitination of and stabilizes its interacting partners to achieve its pathobiological functions (32). As expected, ATXN3 inhibited HIF-2α ubiquitination, which consequently resulted in elevated HIF-2α expression (Figure 3F). Results from further pulse-chase experiments confirmed that ATXN3 expression increased HIF-2α expression levels and prolonged its half-life (Figure 3, G and H). Conversely, ATXN3 targeted suppression resulted in reduced HIF-2α expression and facilitated its degradation (Figure 3, I and J). However, unlike IRF1 and STAT3 coexpression that fully rescued IFN-γ–induced PD-L1 expression (Supplemental Figure 5A), HIF-2α expression could not rescue ATXN3-KO cancer cell expression of PD-L1 when cultivated under hypoxia conditions, nor when IFN-γ was added, suggesting that additional genes are involved in the hypoxia/ATXN3/HIF-2α pathway to control hypoxia-induced PD-L1 expression on cancer cells. Collectively, our results indicate that ATXN3 enhances hypoxia-induced PD-L1 expression through protecting HIF-2α from ubiquitination-induced protein degradation (Figure 3K).

### ATXN3 interacts with multiple transcription factors to promote tumoral PD-L1 expression.

Apart from hypoxia and IFN-γ, several intrinsic oncogenic and tumor microenvironmental factor adaptive pathways are involved in regulating tumoral PD-L1. We then tested the possibility that ATXN3 promotes tumoral PD-L1 expression through downstream transcription factors, such as NF-κB (p65), c-MYC, and AP-1 (Supplemental Table 1). However, ATXN3 interaction with NF-κB (p65), a transcription factor responsible for inflammatory cytokines, such as TNF-α–induced PD-L1 expression, was not detected even when they were overexpressed (Supplemental Table 1). Similarly, the interaction of ATXN3 with c-MYC, a transcription factor responsible for metabolite-induced PD-L1 expression (4), was not detected in transiently transfected HEK293T cells (Supplemental Table 1).

It has been well documented that the AP-1 transcription factor directly promotes PD-L1 expression in cancer cells (33–35). Interestingly, co-IP and Western blot analysis detected the interaction of JunB, but not c-Jun, with ATXN3 in transiently transfected HEK293T cells (Supplemental Figure 6A and Supplemental Table 1), implying a possibility that ATXN3 may enhance AP-1–mediated PD-L1 gene transcription in cancer cells through JunB stabilization. Indeed, the endogenous JunB interaction with ATXN3 was confirmed (Supplemental Figure 6B and Supplemental Table 1), and ATXN3 expression largely abrogated JunB ubiquitination (Supplemental Figure 6C). Consequently, overexpression of ATXN3 dramatically increased JunB protein expression and prolonged JunB half-life (Supplemental Figure 6, D and E, and Supplemental Table 1), indicating that JunB stabilization by ATXN3 may be involved in PD-L1 upregulation in cancer cells. Collectively, our study reveals that ATXN3 is a positive regulator for PD-L1 transcription through stabilizing multiple transcription factors including HIF-2α, IFR1, STAT3, and JunB.

### Suppression of ATXN3 enhances antitumor immunity.

**Figure 4:**
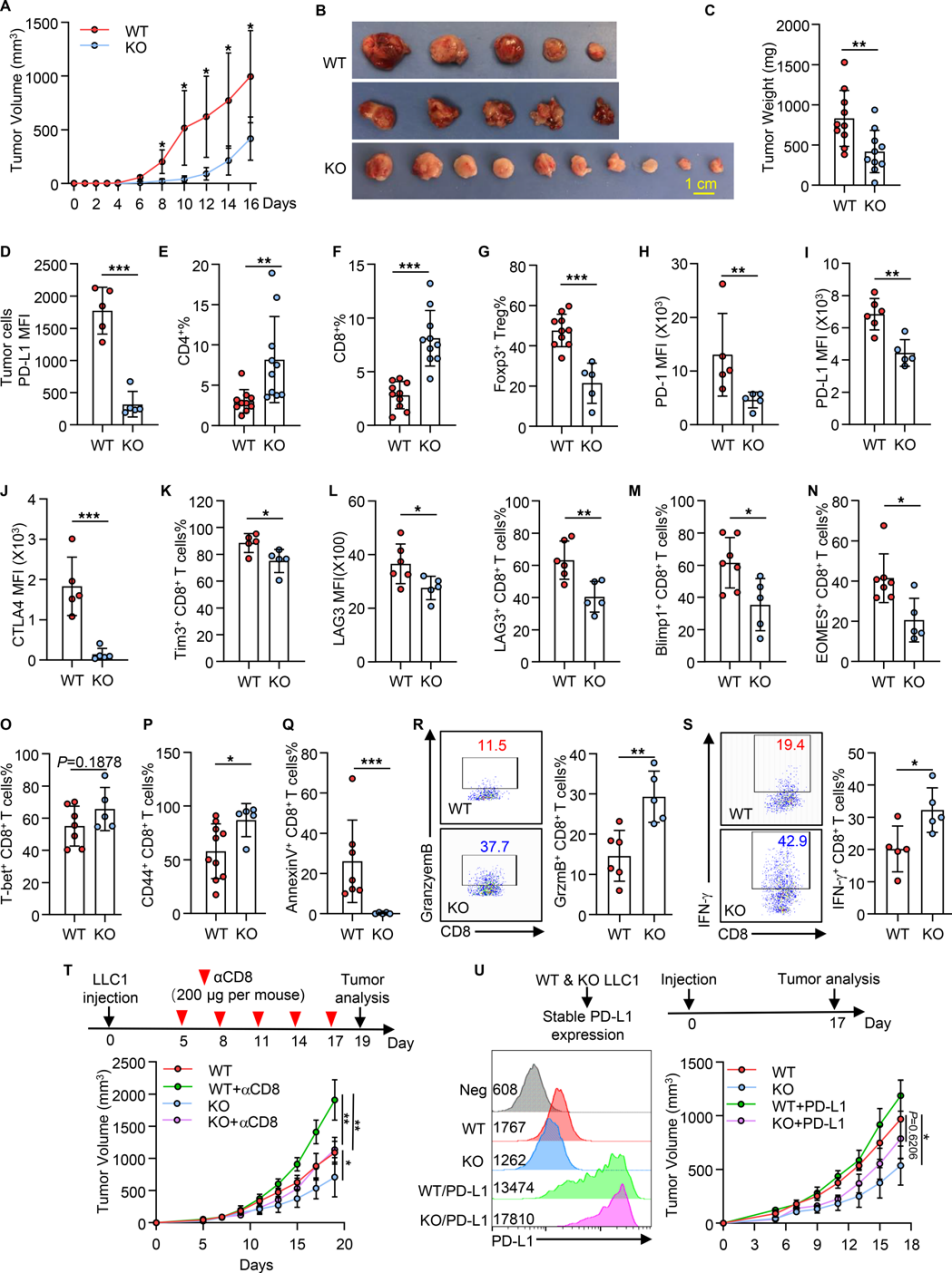
ATXN3 inhibition improves antitumor immunity partially through downregulating tumoral PD-L1 expression. (**A**–**C**) WT or ATXN3-KO LLC1 cells were injected subcutaneously into C57BL/6 mice (*n* = 10). Tumor growth curve (**A**), photograph (**B**), and weight (**C**) are shown. (**D**) MFI of surface PD-L1 on LLC1 tumors (*n =* 5). (**E**–**G**) Quantification of CD4^+^ (**E**) and CD8^+^ T cell (**F**) and Treg (**G**) percentages (*n =* 5–10). (**H** and **I**) Quantification of cell-surface PD-1 (**H**) and PD-L1 (**I**) MFI on CD8^+^ T cells (*n =* 5). (**J** and **K**) Quantification of cell-surface CTLA-4 MFI (**J**) and Tim3 percentage (**K**) in CD8^+^ T cells (*n =* 5). (**L**) MFI of cell-surface LAG3 and percentage in CD8^+^ T cells (*n =* 5). (**M**–**O**) Intracellular staining of Blimp1^+^ CD8^+^ T cell (**M**), EOMES^+^ CD8^+^ T cell (**N**), and T-bet^+^ CD8^+^ T cell (**O**) percentage in LLC1 tumors (*n =* 5-7). (**P**) Quantification of cell-surface CD44^+^ CD8^+^ T cell percentage from LLC1 tumors (*n =* 5–10). (**Q**) Apoptotic CD8^+^ T cells in the tumors were analyzed (*n =* 5–7). (**R** and **S**) Representative flow staining and quantification of intracellular cytokine staining of granzyme B^+^CD8^+^ and IFN-γ^+^CD8^+^ in CD45^+^ T cell populations from LLC1 tumors (*n =* 5). (**T**) Tumor growth curve and tumor photograph of C57BL/6 mice injected subcutaneously with WT and ATXN3-KO LLC1 cells with or without treatment of anti-CD8 depleting antibodies (*n =* 5). (**U**) Left: Tumor cell-surface PD-L1 expression. Right: Tumor growth of WT or ATXN3-KO LLC1 cells stably expressing PD-L1 (as shown in the left plot) in C57BL/6 mice (*n =* 5). **A** and **C**–**S**: 2-tailed unpaired *t* test; **T** and **U**: ordinary 1-way ANOVA. *P < 0.05, **P < 0.01,***P < 0.001.

Tumor cells evade neoantigen-specific antitumor immunity through upregulating their cell-surface expression of checkpoint receptors including PD-L1 (36). Since ATNX3 promotes PD-L1 expression, we posed that ATXN3 suppression may enhance antitumor immunity in vivo. We then used the LLC1 Lewis lung carcinoma syngeneic tumor model to test whether targeted ATXN3 suppression enhances antitumor immunity in C57BL/6 mice. Indeed, upon LLC1 challenge, mice implanted with ATXN3-null LLC1 cells showed striking tumor rejection compared with those with WT LLC1 cells, with a dramatic reduction in both tumor volumes and weight (Figure 4, A–C). Analysis of the PD-L1 expression on CD45^–^ tumor cells confirmed that ATXN3 deletion resulted in a dramatic reduction in PD-L1 expression (Figure 4D). As expected, tumoral ATXN3 deletion resulted in a significant increase in CD4^+^ and CD8^+^ T cell tumor infiltration (Figure 4, E and F). In contrast, the frequency of immunosuppressive FoxP3^+^ Tregs was significantly reduced in ATXN3-KO tumors (Figure 4G). Notably, the expression levels of immunosuppressive receptors including PD-1, PD-L1, and CTLA-4 on the surface of CD8^+^ T cells were all reduced (Figure 4, H–J). Further analysis of tumor-infiltrating CD8^+^ T cells revealed a less exhausted phenotype, with a significant reduction in the fraction of Tim3^+^, LAG3^+^, Blimp1^+^, and EOMES^+^ as well as annexin V^+^ CD8^+^ T cells (Figure 4, K–N and Q), but with a modest increase in T-bet^+^ cells (*P* = 0.1878) and CD44^+^CD8^+^ T cells (Figure 4, O and P). As a consequence, the production of tumor-infiltrating granzyme B and IFN-γ by CD8^+^ T cells in ATXN3-KO tumors was significantly increased (Figure 4, R and S). Further analysis of intratumoral myeloid cells saw comparable frequencies of Gr1^hi^CD11b^+^ myeloid-derived suppressor cells, CD11b^+^F4/80^+^ macrophages, and CD11c^+^MHC-II^hi^ dendritic cells between WT and ATXN3-KO tumors (Supplemental Figure 7, A and B). Furthermore, the expression levels of MHC-I, MHC-II, CD80, and CD86 on myeloid cells were comparable between WT and ATXN3-KO tumors (Supplemental Figure 7C). Similarly, both the frequency and the PD-L1 expression levels of the myeloid cells from tumor-draining lymph nodes were comparable in WT and ATXN3-KO LLC1 tumors (Supplemental Figure 7, D and E). These results indicate that ATXN3 suppression in tumor cells improves antitumor immune response through PD-L1–mediated suppression of CD8^+^ T cell immunity. Indeed, depletion of CD8^+^ T cells partially abolished the tumor growth inhibition caused by ATXN3 knockout (Figure 4T), suggesting that CD8^+^ T cells mediate the elevated antitumor immunity by suppressing ATXN3. Importantly, stable expression of PD-L1 on ATXN3-null LLC1 cells partially recovered the syngeneic tumor growth (Figure 4U), indicating that ATXN3 potentiates tumor evasive function through, at least in part, PD-L1–mediated suppression of antitumor immunity.

Consistently, genetic ATXN3 suppression resulted in reduced PD-L1 expression and better B16 tumor rejection (Supplemental Figure 8, A and B). The elevated tumor rejection by ATXN3 suppression was largely diminished by CD8^+^ T cell depletion (Supplemental Figure 8A), confirming our initial conclusion that tumoral ATXN3 achieves its immune surveillance function in part through suppressing, either directly or indirectly, CD8^+^ T cell antitumor immunity. Flow cytometry analysis of CD45^–^ tumor cells confirmed a dramatic reduction in their surface PD-L1 expression (Supplemental Figure 8B). Further analysis of CD45^+^ intratumoral immune cells detected a statistically significant increase in CD8^+^ but not CD4^+^ T cells (Supplemental Figure 8, C and D); however, the frequency of FoxP3^+^ Tregs was decreased (Supplemental Figure 8E) in ATXN3-KO tumors. Importantly, in addition to their increased frequency, CD8^+^ T cells in ATXN3-null tumors produced significantly higher levers of both IFN-γ and granzyme B (Supplemental Figure 8, F and G). Together with the fact that ATXN3 positively correlated with PD-L1 expression in more than 80% of human cancers, our study collectively shows that ATXN3 inhibition enhances antitumor immunity in a broad spectrum of cancers.

The finding that stable PD-L1 expression could not fully rescue the ATXN3-null syngeneic tumor growth suggests that ATXN3 executes its tumorigenic functions in part through PD-L1–independent mechanisms. However, targeted ATXN3 deletion did not affect LLC1 lung cancer cell growth and colony formation (Supplemental Figure 9, A and B), largely excluding the possibility that ATXN3 promotes LLC1 cancer cell growth. To further support this, we observed that the WT and ATXN3-KO LLC1 tumor growth was comparable in immune-compromised nude mice (Supplemental Figure 9C). In contrast, ATXN3 CRISPR deletion slightly reduced B16 (Supplemental Figure 9, D and E) but increased MC38 cell growth and colony formation in vitro (Supplemental Figure 9, F and G), implying that ATXN3 plays a diverse role in different cancer types. Importantly, targeted deletion of ATXN3 dramatically reduced PD-L1 expression in all types of tumor cells tested (Figure 1, E–H, and Supplemental Figure 2), regardless of whether their proliferation was altered or not. Therefore, our observations collectively support our conclusion that ATXN3-mediated PD-L1 expression is one of the critical mechanisms underlying its tumorigenic functions.

### ATXN3 suppression improves checkpoint blockade antitumor immune therapy.

**Figure 5:**
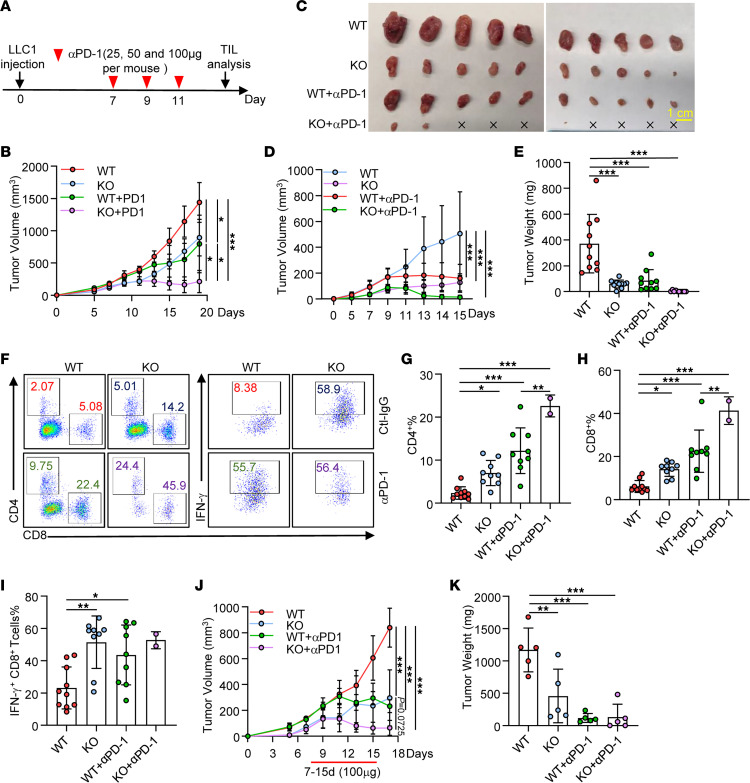
ATXN3 inhibition improves the preclinical efficacy of anti–PD-1 therapy. (**A** and **B**) Scheme representing the experimental procedure (**A**) and tumor growth curves (**B**) of C57BL/6 mice injected subcutaneously with WT or ATXN3-KO LLC1 cells and treated with PD-1 antibody (25 μg per mouse, once every 2 days, *n* = 5). (**C**–**E**) Tumor photograph (**C**), tumor growth curves (**D**), and tumor burdens (**E**) for C57BL/6 mice bearing LLC1 tumors treated with PD-1 antibody (50 μg per mouse, once every 2 days, *n* = 10). (**F**) Representative flow staining of CD4^+^ T cells, CD8^+^ T cells, and IFN-γ^+^CD8^+^ T cells in CD45^+^ T cell populations from LLC1 tumors (*n* = 10) as described in **C** and **D**. (**G**–**I**) Quantification of CD4^+^ T cell (**F**), CD8^+^ T cell (**G**), and IFN-γ^+^CD8^+^ T cell (**H**) percentage in CD45^+^ populations from LLC1 tumors (*n =* 10) as described in **C** and **D**. (**J** and **K**) C57BL/6 mice (6–8 weeks) were injected subcutaneously with WT or ATXN3-KO LLC1 cells and treated with PD-1 antibody (100 μg per mouse, once every 2 days, *n =* 5). Tumor growth curve was measured every 2 days (**J**), and mouse tumors were weighed at the end of the experiment (**K**). **B**, **D**, **E**, and **G**–**K**: Ordinary 1-way ANOVA. *P < 0.05, **P < 0.01, ***P < 0.001.

Tumor cells evade antitumor immunity in part through PD-L1 expression to suppress PD-1^+^ T cell immune response to neoantigens. Therefore, blocking PD-1/PD-L1 binding with specific antibodies enhances antitumor immunity, which has achieved some clinical successes in treatment of human cancers (37, 38). However, checkpoint blockade immunotherapy often causes immune-related adverse events, such as autoimmune inflammatory responses in digestive system, heart, and kidney, which can be lethal (39, 40). Therefore, reducing systemic checkpoint blockade antibodies without impairing therapeutic efficacy has been considered as a future direction. Since ATXN3 suppression reduces tumor PD-L1 expression, we asked whether ATXN3 suppression in tumor cells improves anti–PD-1 therapeutic efficacy. We used a suboptimal dose, 25–100 μg per mouse, of anti–PD-1 antibody for only 3 times to treat pre-established WT and ATXN3-KO syngeneic LLC1 tumors in mice (Figure 5A). Surprisingly, when 25 μg anti–PD-1 antibody was used, we observed a more modest effect on suppressing WT tumor growth, but this dose still largely inhibited ATXN3-KO tumor growth (Figure 5B). Consistently, treatment of mice with ATXN3-null tumors with anti–PD-1 at the suboptimal dose of 50 μg nearly totally rejected the tumor (Figure 5, C–E), implying a synergistic effect of ATXN3 inhibition and anti–PD-1 therapy. Further analysis of tumor-infiltrated immune cells showed a further dramatic increase in both CD4^+^ and CD8^+^ T cells (Figure 5, F–H). Notably, the frequency of IFN-γ–producing CD8^+^ T cells in ATXN3-KO tumors was further increased by anti–PD-1 treatment in mice bearing WT but not ATXN3-null tumors (Figure 5, F and I). Similar results were obtained when mice with WT and ATXN3-KO tumors were treated with a higher dose of anti–PD-1 antibody (Figure 5J), implying that the dose of 50 μg per mouse is sufficient in this syngeneic model. A similar result was obtained when the B16 melanoma model was used (Supplemental Figure 8H), further supporting our conclusion that ATXN3 inhibition enhances checkpoint blockade therapy even with suboptimal anti–PD-1 treatment.

### Positive correlation of ATXN3 with PD-L1 levels in human cancers.

**Figure 6:**
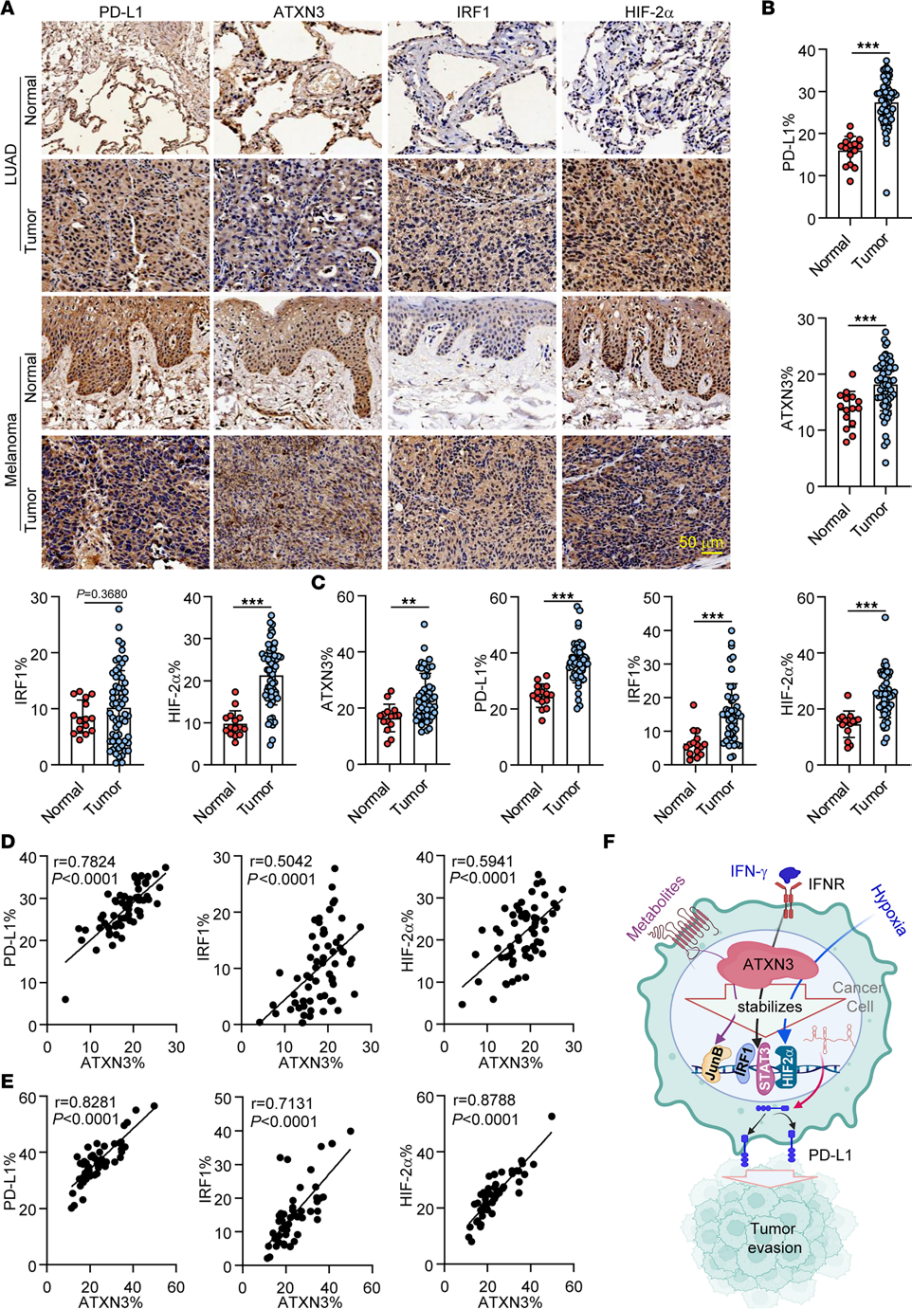
Elevated ATXN3 expression and its positive correlation to PD-L1 and its transcription factors in human lung cancer and melanoma. (**A**) Representative images from immunohistochemical staining of PD-L1, ATXN3, IRF1, and HIF-2α in human lung adenocarcinoma (LUAD) and melanoma patients. Scale bar: 50 μm. (**B**) PD-L1, ATXN3, IRF1, and HIF-2α protein levels in tumor tissues compared with normal tissues in LUAD patients (*n =* 61); “PD-L1%” means the percentage of PD-L1–positive area versus all tissue area. (**C**) PD-L1, ATXN3, IRF1, and HIF-2α protein levels in tumor tissues compared with normal tissues in patients with melanoma (*n =* 48). (**D**) Correlation analysis of ATXN3 expression with PD-L1, IRF1, and HIF-2α expression in LUAD patients (*n =* 61). (**E**) Correlation analysis of ATXN3 expression with PD-L1, IRF1, and HIF-2α expression in melanoma patients (*n =* 48). (**F**) ATXN3 is a positive regulator for PD-L1 transcription through stabilizing multiple transcription factors including HIF-2α, IFR1, STAT3, and JunB and enhances tumor evasion. **B** and **C**: 2-tailed unpaired *t* test; **D** and **E**: Pearson’s correlation analysis. **P < 0.01, ***P < 0.001.

Our data collectively documented that ATXN3 is a positive regulator for PD-L1 transcription and targeted ATXN3 inhibition enhances antitumor immunity. To further validate our findings in human cancers, we analyzed protein expression levels of ATXN3, PD-L1, IRF1, and HIF-2α in human lung adenocarcinoma tissue microarrays and melanoma tissue microarrays, which included 61 lung adenocarcinoma cases and 48 melanoma cases, respectively. Indeed, the protein expression levels of ATXN3, PD-L1, IRF1, and HIF-2α were all elevated in both lung cancer and melanoma compared with their adjacent normal tissues (Figure 6, A–C). Importantly, ATXN3 expression was positively correlated with PD-L1 as well as with the PD-L1 transcription factors HIF-2α and IRF1 (Figure 6, D and E). Therefore, our data support ATXN3 regulation of PD-L1 signaling in human cancer.

Collectively, our study identified ATXN3 as a positive regulator of PD-L1 transcription in tumors through stabilizing a group of PD-L1 transcription factors including HIF-2α, IRF1, STAT3, and JunB in response to extracellular stimuli such as IFN-γ and hypoxia. This ATXN3-mediated PD-L1 upregulation enhances tumor evasion of antitumor immunity (Figure 6F). Therefore, targeted ATXN3 suppression enhances antitumor immunity and improves the preclinical efficacy of antitumor immune therapy.

## Discussion

The current study identifies ATXN3 as a critical positive regulator for tumor invasion through promoting PD-L1 expression at the transcription level and provides a rationale for targeting this deubiquitinase in antitumor immune therapy. This conclusion is documented by the following discoveries: First, ATXN3 was identified as a top activator from our CRISPR screening platform, and targeted ATXN3 deletion by either CRISPR or shRNA resulted in a dramatic reduction in PD-L1 transcription. Second, analysis of the TCGA database revealed a statistically significant positive correlation between *ATXN3* and *CD274* expression in most types of human cancers. Third, ATXN3 promotes PD-L1 transcription in tumor cells through regulating multiple PD-L1 transcription–inducing pathways, including hypoxia and IFN-γ. Fourth, suppression of tumoral ATXN3 dramatically enhanced antitumor immune response to reject the syngeneic LLC1 lung tumors in mice, which is partially dependent on the presence of CD8^+^ T cells, and improved the preclinical efficacy of PD-1 antibody even when administered at a suboptimal dose. Fifth, importantly, reconstitution of PD-L1 expression partially reversed tumor growth of ATXN3-null syngeneic lung cancer. Last but not least, ATXN3 positively correlated with expression of PD-L1 and its transcription factors HIF-2α and IRF1 in both human lung adenocarcinoma and melanoma tissues.

Tumoral PD-L1 expression is regulated by a variety of pathways at both the transcriptional and the posttranslational level. PD-L1 protein can be degraded through ubiquitination by several E3 ubiquitin ligases, including FBXO38, SCFβ-TrCP, HRD1, and STUB1/CHIP, most of which mediate Lys48-linked polyubiquitination and subsequent proteasomal degradation (12, 17–19, 41). On the other hand, several ubiquitin-specific peptidases, including USP7, USP22, OTUB1, and CSN5, regulate PD-L1 protein expression in an opposing fashion through removal of the polyubiquitin modification from PD-L1 and consequently protect PD-L1 from proteasomal degradation in cancer cells (20–23). Using CRISPR-based screening, here we identified ATXN3 as, to our knowledge, the first deubiquitinase that promotes PD-L1 expression at the transcriptional level in cancer cells. Importantly, analysis of the TCGA database showed a statistically significant positive correlation between *ATXN3* and *PD-L1* expression in the majority of types of human cancers, implying that ATXN3 is a positive regulator of PD-L1 in a broad spectrum of human cancers.

Tumor cells often utilize tumor microenvironmental factors to further promote PD-L1 expression to evade antitumor immunity (42). Our study demonstrated that ATXN3 is required for hypoxia- and IFN-γ–induced PD-L1 mRNA transcription through selectively stabilizing their downstream transcription factors including HIF-2α, STAT3, and IRF1. In addition, our biochemistry studies revealed that ATXN3 also functions as a deubiquitinase for the PD-L1 transcription factor JunB. JunB is one of the family members of AP-1. In Hodgkin’s lymphoma, JunB can bind to an enhancer region of the PD-L1 promoter, facilitating PD-L1 expression (33). Under different genotoxic stress, JunB may act as an oncogene or tumor suppressor gene (43); our discoveries here imply that ATXN3 potentiates JunB oncogenic functions including PD-L1–mediated tumor evasion. Moreover, JunB is a critical regulator of IRF4-dependent Treg effector programs and promotes expression of Treg effector molecules, such as ICOS and CTLA-4 (44, 45). The effect of ATXN3 on effector Treg function remains to be further explored.

Since our data show that ATXN3 promotes PD-L1 expression through multiple pathways, it is an interesting question to determine which pathways are predominant. The fact that ATXN3 targeted deletion largely diminished both IFN-γ– and hypoxia-induced PD-L1 expression implies that ATXN3 plays a critical role in promoting PD-L1 expression in inflammatory and hypoxic tumor microenvironment and suggests that ATXN3 regulation of PD-L1 is controlled by extracellular stimuli through distinct pathways. Conversely, in vitro at steady state, targeted deletion of ATXN3 resulted in a relatively modest 20%–40% reduction in surface PD-L1 expression. Notably, a dramatic reduction in PD-L1 by ATXN3 deletion was detected in both LLC1 (more than 90% reduction) and B16 (more than 50% reduction) syngeneic tumors, indicating that ATXN3 plays a critical role in controlling PD-L1 expression in tumor microenvironment. Importantly, coexpression of IRF1 and STAT3 in ATXN3-KO cancer cells fully rescued IFN-γ–induced PD-L1 expression, but had little effect on hypoxia-induced PD-L1 expression, confirming that ATXN3 promotes IFN-γ–induced PD-L1 expression specifically through stabilizing IRF1 and STAT3. However, HIF-2α expression could not rescue ATXN3-KO cancer cell expression of PD-L1 under hypoxia conditions, nor by IFN-γ stimulation, suggesting that additional genes are involved in the ATXN3/HIF-2α pathway to control hypoxia-induced PD-L1 expression. Future studies are needed to further dissect the pathophysiological contacts in ATXN3-mediated PD-L1 expression.

Immune checkpoint inhibitors such as anti–PD-1/PD-L1 blockade antibodies are one of the most important developments in cancer therapy over the past decade (46). However, one cost of these advances is the emergence of a new spectrum of immune-related adverse events, which in many cases can be fatal as a result of severe inflammation in skin, colon, endocrine glands, lungs, kidneys, and liver (47, 48). Development of therapeutics that improves the efficacy of checkpoint blockade immune therapy while reducing immune-related adverse events has been a current focus in the field. The fact that targeted ATXN3 deletion inhibits tumoral PD-L1 expression suggests that ATXN3 suppression could reduce the dose of anti–PD-1 antibodies needed to achieve a favorable therapeutic outcome. In fact, we show here that suboptimal treatment with a lower dose of anti–PD-1 completely inhibited LLC1 syngeneic tumor growth owing to further elevation of the antitumoral immune response, providing a rationale for ATXN3 targeting in sensitizing antitumor immune therapy.

Our data show that depletion of CD8^+^ T cells only partially reverses the antitumor activity of ATXN3 inhibition, suggesting that additional mechanisms are involved in this process. Consistently, PD-L1 reconstitution partially reversed ATXN3 deletion–induced tumor suppression, indicating that additional unknown targets possibly exist for ATXN3 to achieve tumor immune evasive functions. Tumoral PD-L1 has been shown to engage myeloid PD-1 to suppress type I interferon responses and impair cytotoxic T lymphocyte recruitment. Furthermore, myeloid cells are required for PD-1/PD-L1 checkpoint activation and the establishment of an immunosuppressive environment in pancreatic cancer (49, 50). While our flow analysis of intratumoral myeloid cells did not detect any changes in their frequencies and cell-surface expression of MHCs, CD80, and CD86 in ATXN3-null LLC1 tumors, the involvement of myeloid cells, such as their roles in suppressing CD8^+^ T cells, cannot be fully excluded. In addition, ATXN3 has been demonstrated to play a role in maintaining genome stability (51), raising a possibility that targeted suppression of ATXN3 enhances clonal neoantigens in cancer cells. Previous studies have shown that sensitivity to PD-1 blockade in patients with advanced NSCLC was enhanced in tumors enriched for clonal neoantigens; it will be interesting to analyze the effect of ATXN3 suppression on neoantigen presentation as reported (52).

ATXN3 has been reported to play an important role in the development and progression of multiple types of cancers, including breast cancer (53, 54), anaplastic thyroid carcinoma (55), testicular cancer, and non–small cell lung adenocarcinoma (56, 57), in a tumor cell–intrinsic manner. Together with our discovery that ATXN3 drives tumor evasion of immunosurveillance through promoting PD-L1 transcription, targeting of this druggable enzyme will achieve both chemo- and immune-therapeutic efficacy in antitumor treatment. Since ATXN3 promotes PD-L1 expression through STAT3 and HIF-2α, it will be interesting to test whether ATXN3 inhibition synergizes with STAT3- and HIF-2α–specific inhibitors in suppressing tumor growth, which could provide useful insights in translational studies. We are also aware that STAT3, IRF1, and HIF-2α play important roles in immune cells including T cells and myeloid cells, suggesting a potential impact of systemic ATXN3 inhibition in antitumor treatment. While studies report the phenotypic analysis of ATXN3 germline KO mice, which are viable and fertile, there have been no reports of any inflammatory or immune-deficient responses (58). However, the possibility that targeting ATXN3 in non-cancer cells may alter immune functions as a result of accelerated STAT3, IRF1, and HIF-2α degradation cannot be excluded. Future studies are needed to develop ATXN3-specific small-molecule inhibitors for this purpose.

## Methods

### Animal studies

C57BL/6J mice were maintained and used at the Northwestern University mouse facility under pathogen-free conditions. Unless stated otherwise, all figures are representative of experiments with 6- to 8-week-old mice.

For syngeneic mouse tumor models, 5 × 10^5^ cells in 100 μL PBS were subcutaneously injected in the right flank of mice. Starting on day 5 after tumor cell implantation, tumors were measured every 1–2 days (length × width) with a digital caliper. Tumor volume was calculated using the formula: volume = (width^2^) × length/2. All the tumors did not exceed 2,000 mm^3^. For in vivo CD8^+^ T cell depletion, mice received intraperitoneal injection of anti–mouse CD8 antibody (200 μg; Bio X Cell, catalog 53-6.72) or rat IgG2a isotype control (200 μg; Bio X Cell, catalog BE0089) on day 5 after tumor injection and were treated every 3 days. For combined PD-1 therapy experiments, mice received intraperitoneal injection of anti–mouse PD-1 antibody (Bio X Cell, catalog BE0273) or rat IgG2a isotype control (Bio X Cell, catalog BE0089) at a dose of 25 μg, 50 μg, or 100 μg per mouse on day 7 after injection and were treated every 2 days.

### Cell culture, transfection, generation of a stable cell line, and cell treatment

HEK293T (ATCC, CRL-3216), B16 (ATCC, CRL-6322), HCT116 (ATCC, CCL-247), LLC1 (ATCC, CCL-247, with stable expression of OVA), and A549 (ATCC, CCL-185) cells were cultured in DMEM supplemented with 10% FBS. Transfections were performed with Lipofectamine 3000 (Invitrogen, catalog L3000150) according to the manufacturer’s instructions. Forty-eight hours after transfection, cells were harvested and subjected to various assays. For gene knockout or knockdown, cells were selected in the presence of puromycin (MedChemExpress, catalog HY-B1743Aa) for at least 2 days to generate stable cell lines. For cell degradation experiments, the transfected HEK293T cells or A549 cells were treated with cycloheximide (Cell Signaling Technology, catalog 2112) for different time intervals.

To establish the hypoxia model, cells were incubated in a microaerophilic system (Thermo Fisher Scientific) with 5% CO_2_ and 1% O_2_ balanced with 94% N_2_ gas for 24 hours. For IFN-γ activation experiments, A549 cells were plated in 24-well plates and, the following day, treated with recombinant human IFN-γ (10 ng/mL; PeproTech, catalog AF-300-02) for 24 hours.

### Development of deubiquitinase CRISPR screening

#### Deubiquitinase plasmid library.

To generate our lentiviral CRISPR library, we used the lentiCRISPR v2 vector system. Guides targeting a total of 96 mammalian deubiquitinase family members were picked from the Mouse GeCKO v2 CRISPR knockout library B subset (59) (Supplemental Table 2). Three guides were picked to target each gene, and 10 non-targeting guides were included in the library (24, 59). The lentiCRISPR v2 system is a single-vector system that contains the sgRNA sequence, Cas9, and puromycin resistance cassettes (24, 59).

SgRNA guide inserts were chemically synthesized (IDT) and cloned individually per the protocol described by Sanjana et al. (59). Briefly, the lentiCRISPR v2 plasmid was cut with BsmBI (FastDigest, Thermo Scientific) followed by annealing of each sgRNA oligonucleotide. Plasmids were transformed and amplified in chemically competent *E*. *coli* (Lucigen Endura) and purified using an endotoxin-free PureLink HiPure Plasmid Miniprep Kit (Invitrogen, catalog K210003). A total of 298 plasmids were cloned and the guide plasmids were pooled at equal concentrations to generate the complete library, which was further validated by sequencing.

#### Lentivirus infection and multiplicity of infection.

The lentiviral library was generated using the DUB plasmid library and the second-generation packaging plasmids psPAX2 and pMD2.G per Sanjana et al.’s protocol (24, 59). Pooled plasmids were cotransfected with packaging plasmids into HEK293T cells using the jetPRIME Transfection reagent (Polyplus, catalog 117-15). Viral supernatant was harvested 48–72 hours after transfection. Lenti-X GoStix kit (Takara, catalog 631280) was used to estimate lentivirus titer by quantifying lentiviral p24 antigen.

To determine the multiplicity of infection (MOI) for B16 melanoma cells, we generated a vector control lentivirus as described above containing the lentiCRISPR v2 plasmid in place of the pooled library. Functional lentivirus titer for B16 cells was identified by infection with the vector lentivirus at various titers. Cells were then subjected to puromycin selection at 2 μg/mL 48 hours after transduction followed by quantification of colony-forming units to identify functional titer. To further confirm lentivirus infection, we stained for Cas9 expression in B16 cells infected with vector lentivirus. Three days after transduction, cells were stained in PBS supplemented with 2% FBS (FACS Buffer) with intracellular Cas9 (Alexa Fluor 488 Conjugate) at 1:50 concentration (Cell Signaling Technology, catalog 7A9-3A3) along with a fixable viability dye (eBioscience eFluor 450) (Invitrogen, catalog 65-0863-14) at 1:1,000 concentration. Cells were run on a BD LSRFortessa Cell Analyzer (BD Biosciences), and the mean fluorescence intensity (MFI) of Cas9 was analyzed using FlowJo (Tree Star).

#### CRISPR B16 FACS.

Once the concentration of lentivirus needed for an MOI of 0.3 was determined, B16 cells were transduced at an MOI of 0.3 with the pooled DUB-KO lentivirus library. Forty-eight hours after transduction, 2 μg/mL of puromycin was added for 3–4 days alongside a non-transduced control to determine duration of selection. Cells were then kept in culture for 3–4 days before sorting. To prepare cells for sorting, B16 cells were harvested using Accutase (Corning, catalog 25-058-CI), stained in FACS Buffer with Fixable Viability Dye (1:1,000) and PD-L1–APC (BioLegend, catalog 124312) at a concentration of 1:100, and sorted on a BD FACSAria (BD Biosciences). Cells were sorted on high PD-L1 expression (top 5% PD-L1 MFI) and low PD-L1 expression (bottom 5% PD-L1 MFI). A minimum of 150,000 cells of each population was sorted to maintain a coverage of at least 500 cells per guide. Sorted cells were pelleted and flash-frozen. Genomic DNA was extracted along with the addition of Proteinase K treatment (Zymo Research).

#### Guide library preparation and sequencing.

Primers for sequencing library preparation were adapted from Sanjana et al.’s protocol (24, 59) and synthesized by IDT. Genomic DNA was prepared for sequencing in a 1-step PCR as reported (24). All primers used for this study are listed in Supplemental Table 3. Post-PCR sample libraries were purified by running through a 2% agarose gel followed by gel excision and extraction (Qiagen, catalog 28704). Library quality was verified on a Bioanalyzer (Agilent) and sent for Illumina Next Generation Sequencing using a MiSeq at the Northwestern Sequencing Core Facility. The original DUB plasmid library was also included as a separate sample in the sequencing run to verify appropriate distribution of individual guides. Sequencing reads were processed using FastQC and MultiQC packages followed by analysis with MAGeCK (60) count and test functions to generate summary statistics for each guide as well as a radioreceptor assay enrichment score to rank sgRNAs that were positively selected in each treatment group based on *P* values. sgRankView from the MAGeCKFlute package was used to visualize the log fold change in guides per gene (60, 61). Guide distribution in the library was verified using the plasmid pool, and non-represented guides were dropped from analysis. Samples were normalized to the median read distribution followed by comparison with the original plasmid pool.

### Plasmids and other reagents

ShRNA sequences for human *ATXN3*, CGTCGGTTGTAGGACTAAATA (shRNA405, catalog TRCN0000007405) and GCAGGGCTATTCAGCTAAGTA (shRNA407, catalog TRCN0000007407), were purchased from Sigma-Aldrich. HA-ubiquitin, FLAG-IRF1, c-Jun, and STAT3 expression plasmids were used as reported (62, 63). HA–HIF-2α (catalog 18950), HA–HIF-1α (catalog 18949), NF-κB (p65) (catalog 20012), JunB (catalog 29687), and c-MYC (catalog 16011) were purchased from Addgene. Plasmids expressing human deubiquitinase were provided by Lingqiang Zhang from the Beijing Institute of Radiation Medicine or purchased from Addgene.

### Flow cytometry analysis of membrane PD-L1

For cultured cell lines, cells were digested with Accutase solution and collected by centrifugation at 350xg for 5 minutes. The cells were stained along with a fixable viability dye (eBioscience eFluor 450) and PE-conjugated PD-L1 antibody (Invitrogen, catalog 12598342; BioLegend, catalog 124308) at 4°C for 30 minutes in the dark. After washing with FACS Buffer, the cells were acquired using a BD flow cytometer, and data were analyzed using FlowJo software.

### Tumor-infiltrating lymphocyte analysis

Mice were euthanized by CO_2_ narcosis followed by cervical dislocation. Tumors were removed, weighed, and dissociated using 3–5 mL digestion medium (4 mg/mL collagenase IV; Worthington Biochemical, catalog LS004188). Samples were then mashed and filtered to produce a single-cell suspension. Cells were stained with Fixable Viability Dye eFluor 450 (Invitrogen, catalog 65-0863-14), Alexa Fluor 700–CD45 (BioLegend, catalog 109821), BV785-CD4 (BioLegend, catalog 100453), BV510-CD8 (BioLegend, catalog 100751), PE-CD25 (BioLegend, catalog 102008), PE-Cy7–CD44 (BioLegend, catalog 103030), BV510–PD-1 (BioLegend, catalog 135241), PE-LAG3 (eBioscience, catalog 12-2231-81), and APC-Tim3 (eBioscience, catalog 17-5871-82) for 30 minutes on ice. The stained cells were then washed with FACS Buffer. For intracellular staining, cells were fixed and permeabilized using reagents from BioLegend (catalog 00-8333-56, 00-5123-43) and stained with specific antibodies against FITC-FoxP3 (eBioscience, catalog 11-5773-82), PE-Cy7–T-bet (eBioscience, catalog 255825-82), PE-Blimp1 (BD Biosciences, catalog 564268), PE-Cy7–EOMES (eBioscience, catalog 25-4875-82), and PE-Cy7–CTLA-4 (BioLegend, catalog 106313).

For intracellular cytokine staining, cells were stimulated with PMA (10 ng/mL; Sigma-Aldrich, catalog P1585), ionomycin (1 mg/mL; Sigma-Aldrich, catalog I0634), and monensin (1:1,000; BioLegend, catalog 420701) for 4 hours, and finally stained with FITC–granzyme B (BioLegend, catalog 515403) antibody and PE-Cy7–IFN-γ (BioLegend, catalog 505826) antibody. Annexin V staining was performed using an annexin V binding buffer (BioLegend, catalog 42201) and stained with APC–annexin V (BioLegend, catalog 640920) and PI (BioLegend, catalog 79997) antibody. Subsequently, cells were flowed on the BD flow cytometer, and data were analyzed using FlowJo software.

### Generation of stable PD-L1–overexpressing cell lines

To generate LLC1 cells wherein PD-L1 is stably overexpressed, we subcloned the coding sequence of PD-L1 (Origene, catalog MC201908) into a pCMV-GFP lentiviral vector using 2 restriction enzymes, NheI and EcoRI, with a double digest protocol. The empty vector was used as a negative control. The PD-L1 expression construct was cotransfected with packaging plasmids into HEK293T cells using the jetPRIME Transfection reagent. After 48 hours of incubation, the packaged lentiviruses were collected and used to infect LLC1 cells. After 2 days, stable cells (GFP^+^) were selected by fluorescence-activated cell sorting.

### Coimmunoprecipitation and Western blotting

Cells were washed with ice-cold PBS, lysed in RIPA lysis buffer with protease inhibitor, and incubated on ice for 15 minutes, followed by centrifugation at 15,000*g* for 15 minutes. Supernatants were pre-cleaned with Protein G Sepharose (GE Healthcare, catalog 17-0618-02) 3 times for 15 minutes each time and subjected to immunoprecipitation with each indicated antibody, then incubated for 2 hours on ice followed by the addition of 50 μL of Protein G Sepharose beads for 2 hours. The beads were then washed 5 times and boiled with 50 μL of 2× loading buffer for 5 minutes, and proteins were separated on 8%–10% SDS-PAGE gels and transferred to nitrocellulose membranes. The membranes were blocked in 5% fat-free dried milk in Tris-buffered saline with 0.5% Tween-20 (TBST) for 1 hour. The membranes were then incubated with the appropriate primary antibodies overnight at 4°C. Membranes were washed in TBST and then incubated in horseradish peroxidase–conjugated (HRP-conjugated) secondary antibodies (MilliporeSigma, goat anti-rabbit IgG antibody, HRP conjugate, catalog 12-348; goat anti-mouse IgG antibody, HRP conjugate, catalog 12-349) for 1 hour. Then membranes were washed in TBST, and the signals were visualized using enhanced chemiluminescence substrate (Thermo Fisher Scientific, catalog 34577) and quantified using Bio-Rad Image software. When necessary, membranes were stripped using stripping buffer (Thermo Fisher Scientific, catalog 46430) and reincubated with corresponding antibodies. Primary antibodies used were as follows: ATXN3 (Proteintech, catalog 67057-1-Ig), HIF-2α (Abcam, catalog ab207607), PD-L1 (Abcam, catalog ab213480; Cell Signaling Technology, catalog 13684S), β-actin (Proteintech, catalog 66009-1-Ig), GAPDH (Proteintech, catalog 10494-1-AP), and FLAG-tag (MilliporeSigma, catalog F1804). Antibodies against HA-tag (catalog 3724S), Myc-tag (catalog 2278S), IRF1 (catalog 8478), STAT3 (catalog 9139), STAT1 (catalog 14994T), JunB (catalog 3753), NF-κB (p65) (catalog 6956), c-Jun (catalog 9165), and c-MYC (catalog 9402) were purchased from Cell Signaling Technology.

### Luciferase reporter assay

HEK293T cells were plated in 96-well plates and, the following day, cotransfected with 0.01 μg TK control (*Renilla* luciferase), 0.05 μg ATXN3 plasmid, and 0.05 μg *CD274* promoter reporter (firefly luciferase, Addgene, 107007) constructs using Lipofectamine 3000 (Invitrogen, catalog L3000150). After 48 hours, the luciferase activity was assessed using the Dual-Luciferase Reporter reagent (Promega, catalog E2940) according to the manufacturer’s instructions. The relative firefly luciferase activity was normalized to *Renilla* luciferase activity, and fold change was normalized to the control value. In a dose-dependent manner, luciferase activity of *CD274* reporter in HEK293T cells was assessed after cotransfection with different doses (0, 0.0125, 0.025, 0.05, 0.1, 0.2 μg) of ATXN3 plasmid.

### Real-time quantitative PCR with reverse transcription

Total RNA was extracted using TRIzol Reagent (Invitrogen, catalog 15596018) or the RNeasy Micro Kit (Qiagen, catalog 74106) and then reverse-transcribed with the qScript cDNA synthesis kit (Quanta Bioscience, catalog 84003). Quantitative PCR was performed using the qScript cDNA Synthesis kit (Quanta Biosciences, catalog 95047-100). The mRNA level was calculated using the ΔCt method and normalized by β-actin. Primers for mouse or human genes, including β-actin, ATXN3, and CD274, were purchased from Real Time Primers. All primers used for this study are listed in Supplemental Table 3.

### Immunohistochemical staining of tissue microarrays

Tumor tissue microarrays, purchased from Bioaitech Co. Ltd. (catalog R076Lu01, K063Me01), contained 61 lung adenocarcinoma cases and 48 melanoma cases. Paraffin-embedded human tissue microarrays were deparaffinized, rehydrated, subjected to heat-induced antigen retrieval, blocked in goat serum blocking solution at room temperature for 30 minutes, and then incubated with primary antibodies overnight at 4°C. The next day, the sections were washed and then incubated with biotin-conjugated secondary antibodies for 1 hour at room temperature, followed by DAB chromogenic reaction. Hematoxylin staining solution was used for nucleus counterstaining. The images were captured using a Nikon microscope and analyzed by Aipathwell software. Primary antibodies used for immunohistochemistry were antibodies against PD-L1 (1:200; Cusabio, catalog CSB-MA878942A1m), ATXN3 (1:500; Proteintech, catalog 13505-1-AP), IRF1 (1:100; Cell Signaling Technology, catalog 847S), and HIF-2α (1:100; Thermo Fisher Scientific, catalog PA1-16510). The percentage of positive area indicates positive area versus all tissue area.

### Statistics

Statistical analysis was carried out using GraphPad Prism 8 software, and tests used for each experiment are listed in the figure legends. Statistical significance was analyzed using the unpaired, 2-tailed Student’s *t* test and 1-way ANOVA test. Pearson’s correlation analysis was performed to determine the correlation between 2 variables. Results are presented as mean ± SD of 3 independent experiments; *P < 0.05, **P < 0.01, ***P < 0.001.

### Study approval

The use of animals for the current study was reviewed and approved by the Northwestern University institutional animal care and use committee (IACUC). All animal experiments followed Northwestern University IACUC approved protocol IS00015611. All animal procedures were performed in accordance with the NIH *Guide for the Care and Use of Laboratory Animals* (National Academies Press, 2011) as detailed in the protocol. While commercial human cancer tissue microarray slides were used, no IRB review was required for this study.

### Data availability

Values for all data points found in graphs can be found in the Supporting Data Values file. All raw, uncropped Western blots are available as supplemental material. Additional details regarding data and protocols that support the findings of this study are available from the corresponding author upon request.

## Author contributions

SW, RI, and MY performed the studies and analyzed the data. NL and YC assisted with cell work and performed some experiments. XH, YZ, and QG provided patient tissue samples. XH and LZ contributed critical reagents. JW constructed deubiquitinase plasmids. MY, ZS, and DF designed the study and wrote the manuscript.

## Supplementary Material

Supplemental data

Supporting data values

## Version 1 12/01/2023

Electronic publication
