# Supplementary material for: CRISPR screening identifies the deubiquitylase ATXN3 as a PD-L1–positive regulator for tumor immune evasion
Source: J Clin Invest. 2023 Dec 1;133(23):e167728. doi: 10.1172/JCI167728 (PMC10688982; doi:10.1172/JCI167728)
Supplement: Supplemental data [file jci-133-167728-s021.pdf]

CRISPR screening identifies the deubiquitylase ATXN3 as a PD-L1 positive regulator for tumor immune evasion

Shengnan Wang[1,2], Radhika Iyer[2], Xiaohua Han[3], Juncheng Wei [2], Na Li[1], Yang Cheng[2], Yuanzhang Zhou[1], Qiong Gao[1], Lingqiang Zhang [4], Ming Yan[2, 5], Zhaolin Sun[1] and Deyu Fang[2]

Supplemental Figures 1-9 & legends
Supplemental Table 1-3

Fig. s1

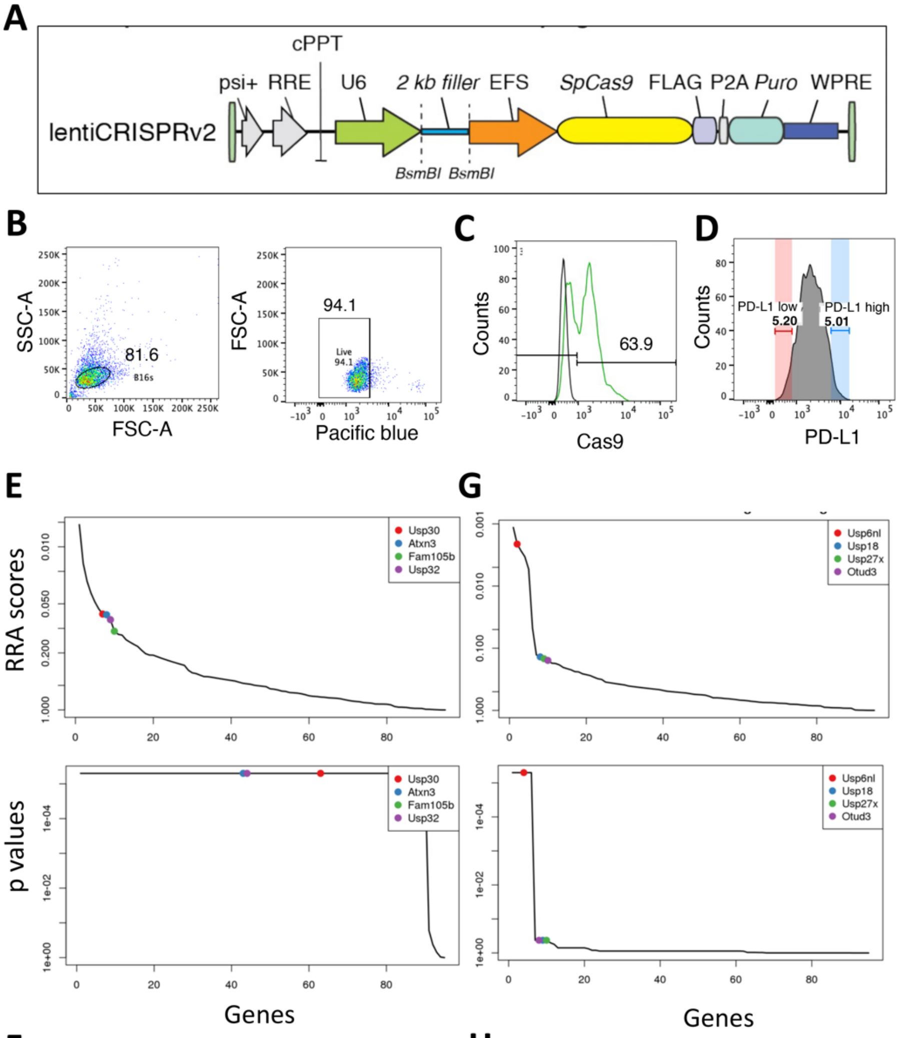

F

sgrna	Gene	LFC	p.value
ID_186	Atxn3	-1.3575	0.00157615
ID_109	Usp32	-0.89494	0.00218605
ID_110	Usp32	-0.43108	0.00384735
ID_103	Usp30	-0.062128	0.0060405
ID_185	Atxn3	1.2435	0.015624
ID_111	Usp32	1.5177	0.0134625
ID_105	Usp30	1.4316	0.012835
ID_232	Fam105b	1.4038	0.010844
ID_184	Atxn3	0.62077	0.0079385
ID_233	Fam105b	0.46874	0.005583
ID_234	Fam105b	0.45027	0.005567

H

sgrna	Gene	LFC	p.value
ID_18	Usp6nl	3.4404	0.00328175
ID_17	Usp6nl	3.07	0.017643
ID_206	Otud3	-1.7248	0.05714
ID_71	Usp18	0.15768	0.22673
ID_72	Usp18	0.46838	0.282845
ID_94	Usp27x	0.76408	0.32805
ID_16	Usp6nl	0.84514	0.34083
ID_95	Usp27x	0.8243	0.35782
ID_207	Otud3	1.0073	0.40421
ID_70	Usp18	1.5072	0.45178
ID_96	Usp27x	1.4746	0.46005
ID_205	Otud3	1.2811	0.491285

Figure S1. CRISPR screening for PD-L1 deub regulators. (A) Schematic showing the lentiCRISPRv2 vector system used to generate the CRISPR-KO library. **(B-D)** Gating strategy for live cells (B). Representative Flow Cytometry plots confirming lentivirus infection of B16 cells through intracellular staining of Cas9 (C). Representative FACS plots from sorting. B16 cells were sorted on live cells followed by their PD-L1 expression. Top and bottom 5% of PD-L1 MFI populations were gated and sorted (D). **(E-H)** The RRA scores, p values and folds of enrichment of the identified guides are shown.

Fig. s2

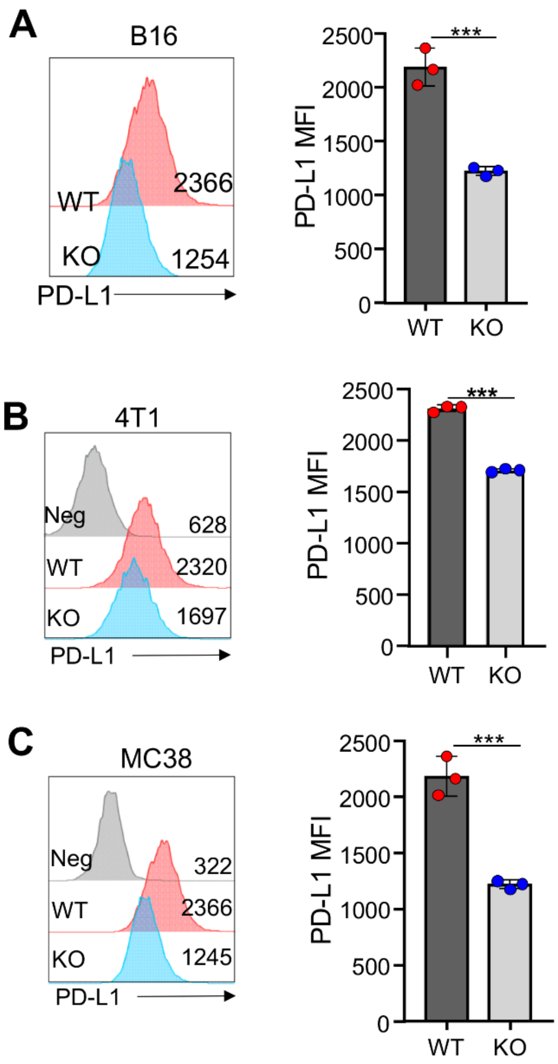

Figure s2. Analysis of PD-L1 expression on WT and ATXN3 KO cells. The expression levels of PD-L1 on WT and ATXN3 KO B16 melanoma **(A)** 4T1 triple-negative breast cancer **(B)** and MC38 colon cancer **(C)** cells was analyzed by flow cytometry. Representative images (left) and data from 3 repeated experiments (right) are shown. Two-tailed unpaired t test was performed to determine statistical significance. *** $P < 0.001$.

Fig. s3

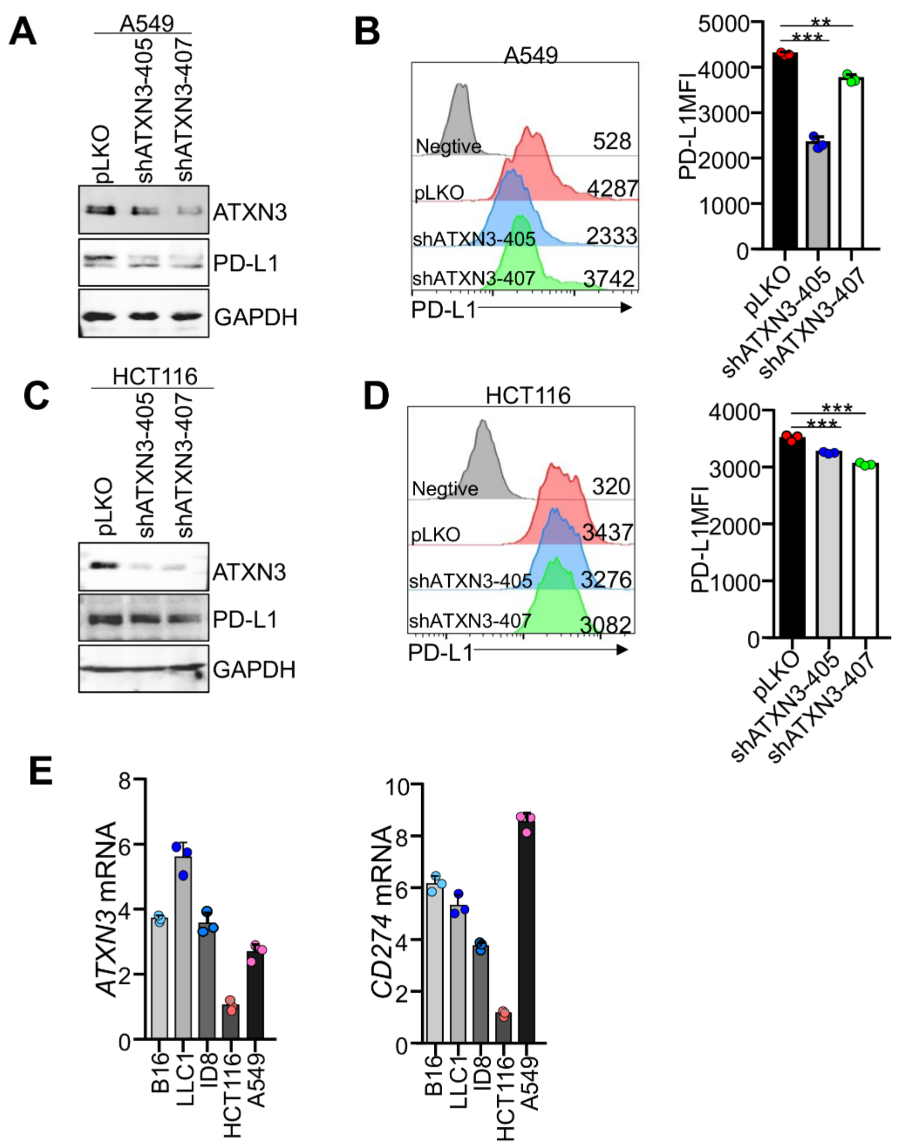

Figure S3. (A) PD-L1 protein expression in A549 cells with ATXN3 knockdown was detected by western blotting. **(B)** Representative flow cytometry plots and quantification of cell-surface PD-L1 in ATXN3 knockdown A549 cells. **(C)** PD-L1 protein expression in HCT116 cells with ATXN3 knockdown was detected by western blotting. **(D)** Representative flow cytometry plots and quantification of cell-surface PD-L1 in ATXN3 knockdown HCT116 cells. **(E)** *CD274* and *ATXN3* mRNA levels were analyzed by reverse transcription quantitative PCR (RT-qPCR) inB16, LLC1, ID8, HCT116 and A549 cells. (B and D) Ordinary one-way ANOVA with multiple comparisons was performed to determine significance. *$P < 0.05$, ** $P < 0.01$, *** $P < 0.001$.

Fig. s4

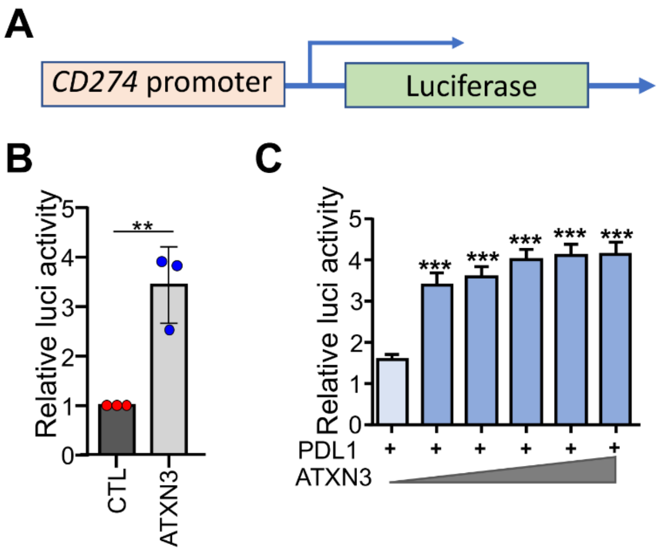

Figure s4. **(A)** Schematic showing modified Luciferase expression vector with luciferase expression under control of *CD274* promoter used in (B-C). **(B)** Effect of ATXN3 on PD-L1 transcription was analyzed through dual-luciferase reporter assays. **(C)** Luciferase activity of PD-L1 reporter in HEK293T cells after co-transfection with different doses of ATXN3 plasmid. Ordinary one-way ANOVA with multiple comparisons was performed to determine significance. **P*<0.05, ***P*<0.01, ****P*<0.001.

Fig. s5

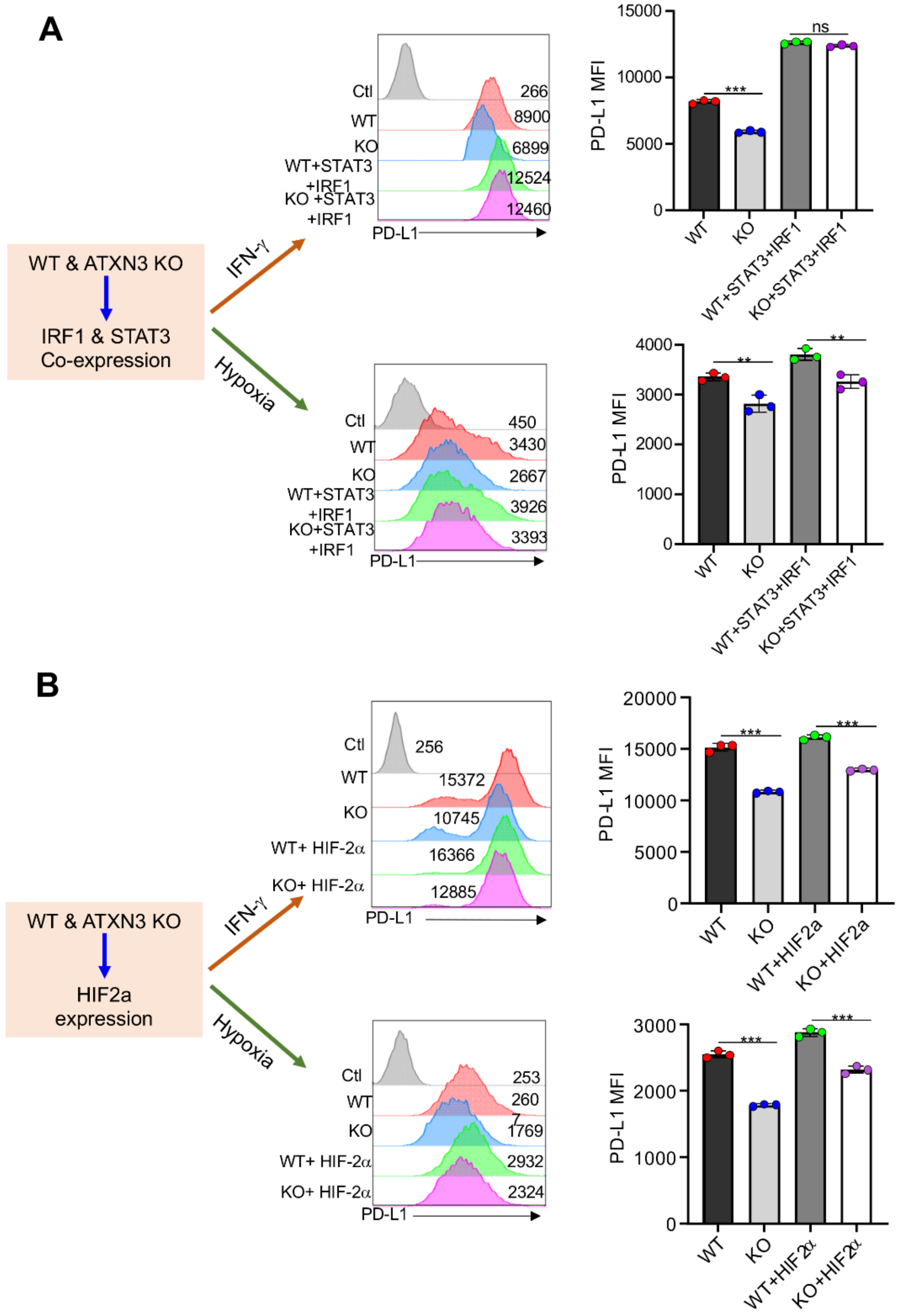

Figure s5. The effect of IRF1/STAT3 or HIF2a expression on PD-L1 expression in ATXN3 WT and KO LLC1 cells. WT or ATXN3 KO LLC1 cells were transfected with IRF1 and STAT3 **(A)** or with HIF-2a **(B)**. 36 hours later cells were cultivated either with IFN-g or under hypoxia condition for additional 24 hours and their surface PD-L1 expression were analyzed by flow cytometry. Ordinary one-way ANOVA with multiple comparisons was performed to determine significance. $*P < 0.05$, $** P < 0.01$, $*** P < 0.001$.

Fig. s6

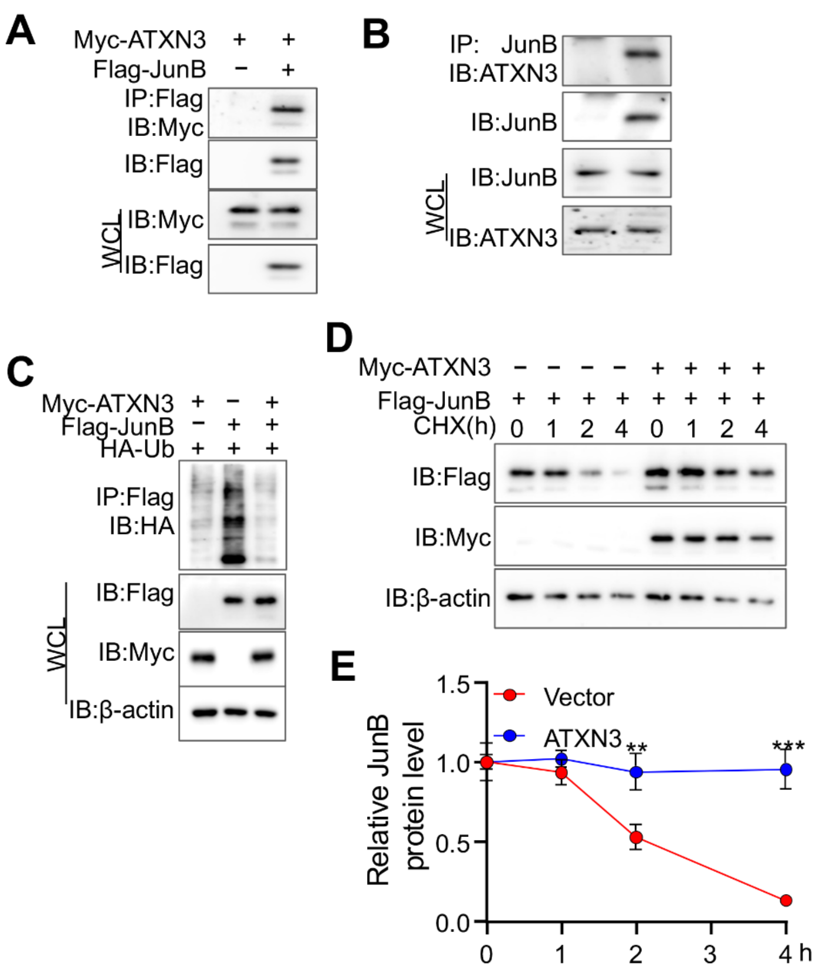

Figure s6. ATXN3 is a JunB deubiquitinase. (A) ATXN3 interacts with JunB. Myc-ATXN3 expression plasmid was co-transfected with or without Flag-JunB into HEK293T cells. JunB protein was immunoprecipitated with anti-Flag antibody, bound ATXN3 was detected with anti-Myc antibody (top panel). **(B)** The endogenous interaction between ATXN3 and JunB in A549 cells. **(C)** HA-Ub and Flag-JunB expression plasmids were cotransfected with Myc-ATXN3 into HEK293T cells. JunB ubiquitination was determined by immunoprecipitation of JunB with anti-Flag antibodies and immunoblotting with HA antibody. **(D & E)** Flag-JunB was cotransfected with or without Myc-ATXN3 plasmids into HEK293T cells. The transfected cells were treated with cycloheximide (CHX) for different times. The protein levels of JunB (top panel) and ATXN3 (middle panel) were analyzed by western blotting. β-actin was used as a loading control (bottom). (E) Two-tailed unpaired t test was performed to determine statistical significance.*P<0.05, **P<0.01, ***P<0.001.

Fig. s7

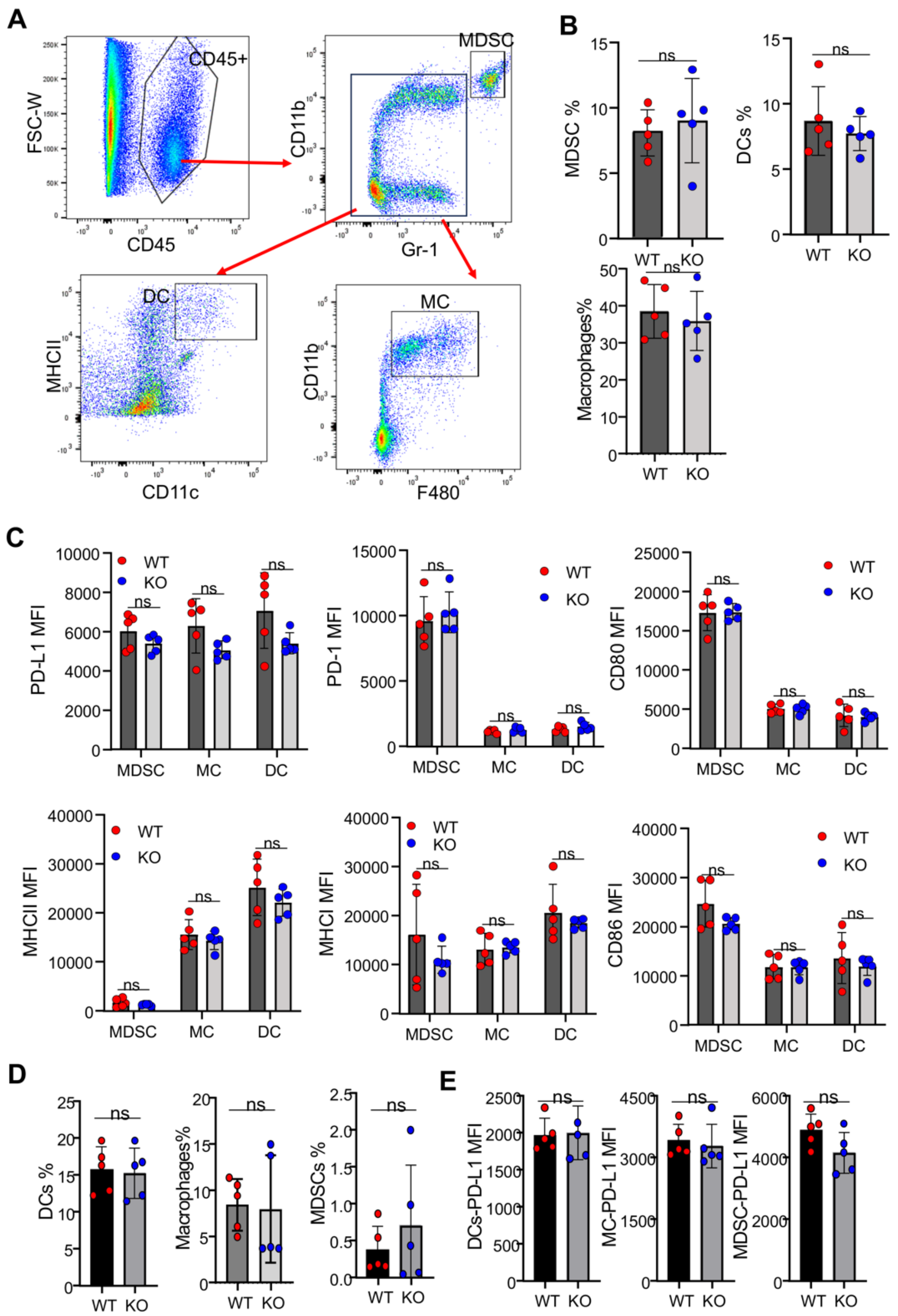

Figure s7. Analysis of the intratumoral myeloid cells in WT and ATXN3 KO tumors. (A) CD45+ intratumoral cells were further analyzed using CD11b, Gr1, F4/80, CD11C and MHC-II. **(B & C)** The frequencies of Gr1^{hi}CD11b^{+} MDSCs (myeloid-derived suppressor cells), CD11b^{+}F4/80^{+} macrophages (MCs) and CD11c^{+}MHC-II^{hi} dendritic cells (DCs) and their surface expression of MHC-I, MHC-II, CD80, CD86 and PD-L1 are analyzed. **(B & C)** Analysis of the frequency and their PD-L1 expression of myeoloid cells from tumor draining lymph nodes. (B-E)

Two-tailed unpaired t test was performed to determine statistical significance.*P<0.05, **P<0.01, ***P<0.001.

Fig. s8

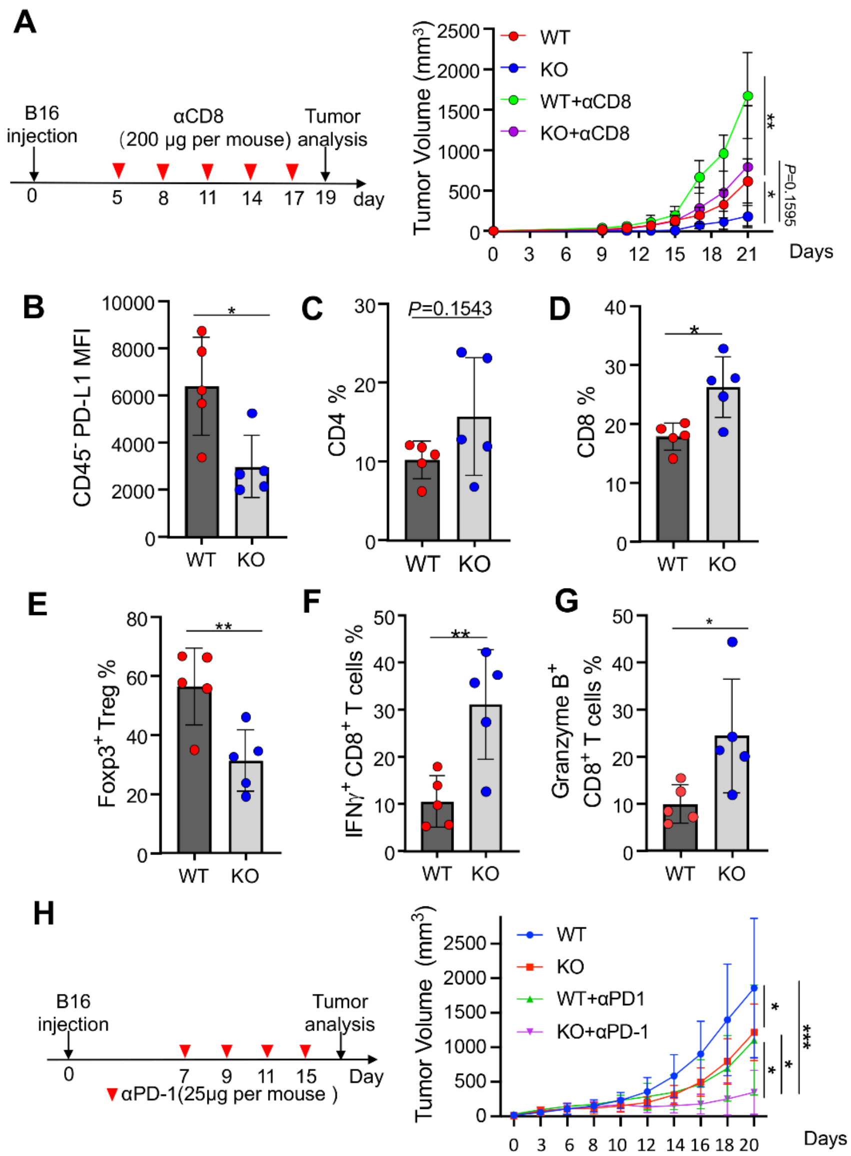

Figure s8. ATXN3 inhibition improves anti-B16 melanoma immunity partially through downregulating tumoral PD-L1 expression. (A) WT or ATXN3 KO B16 melanoma cells were injected subcutaneously into C57BL/6 mice and then treated with or without anti-CD8 depletion Ab as indicated. Tumor growth curve was measured every 2 days. **(C)** Quantification of $CD45^-$ cell-surface PD-L1 MFI from LLC1 tumors (n = 5). **(D-H)**, Quantification of $CD4^+$ T cells (D), $CD8^+$ T cells (E) and Treg cells (F) percentage in $CD45^+$ populations from B16 tumors as well as the CD8 T cell production of IFN-γ (G) and Granzyme B (H) were analyzed. (A), **(I)** WT or ATXN3 KO B16 melanoma cells were injected and then treated with anti- PD-1 as indicated. (A

and H) Ordinary one-way ANOVA with multiple comparisons was performed to determine significance. (B-G) Two-tailed unpaired t test was performed to determine statistical significance. *$P < 0.05$, ** $P < 0.01$, *** $P < 0.001$.

Fig s9

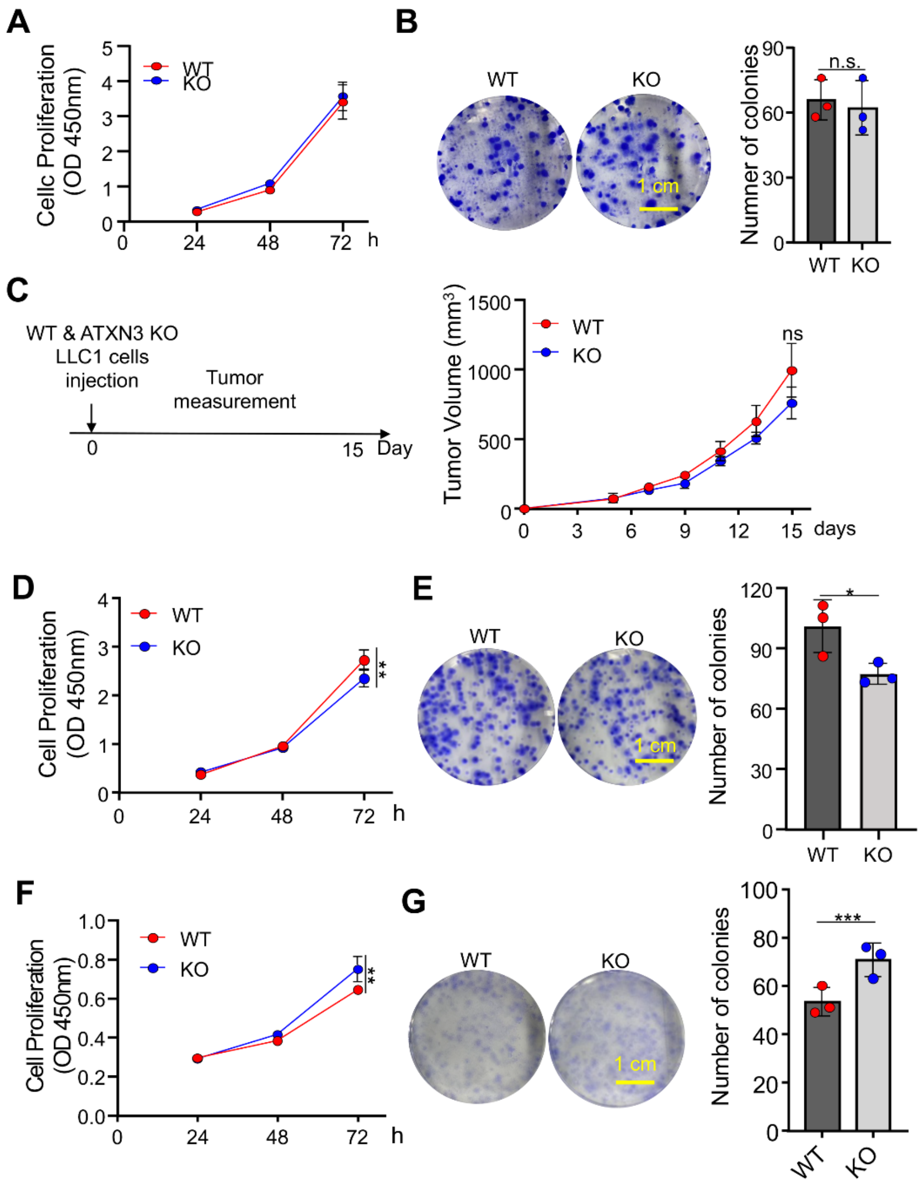

Figure s9. The effects of ATXN3 knockout on cancer cell growth. (A-C) The effect of *Atxn3* knockout on LLC1 proliferation (A) and colony formation (B) in vitro were determined by WST-1 reagent and argar culture, respectively. The tumor progression of WT and ATXN3-KO LLC1 tumors in immune comprimized mice (C). **(D-G)** The effect of *Atxn3* knockout on B16 (C & D) and

MC38 (F & G) cell proliferation (D & F) and colony formation (E & G) in vitro were determined by WST-1 reagent and argar culture, respectively. *$P < 0.05$, ** $P < 0.01$, *** $P < 0.001$. Two-tailed unpaired t test was performed to determine statistical significance.*$P<0.05$, **$P<0.01$, ***$P<0.001$.

Table 1. ATXN3 interacts with multiple transcription factors to promote tumoral PD-L1 expression.

PD-L1 TFs	Interaction	Endogenous Interaction	Deubiquitination	Degradation
NF-κB	-	NT	NT	NT
IRF1	+	+	+	+
JunB	+	+	+	+
c-JUN	-	NT	NT	NT
c-MYC	-	NT	NT	NT
HIF-2α	+	+	+	+
HIF-1α	-	NT	NT	NT
STAT3	+	+	+	+
STAT1	-	-	NT	NT

+ positive; - negative; TF, transcription factor; NT, not tested.

Suppl Table 2 Guide sequences for all 96 deubiquitinase family members

Alg13	ATCTTGTGGAAAGGACGAAACACCGGAACAATCATCAATTTGAATGTTTTAGAGCTAGAAATAGCAAGTT
Alg13	ATCTTGTGGAAAGGACGAAACACCGGATCTTGTCATTAGCCACGCGTTTTAGAGCTAGAAATAGCAAGTT
Alg13	ATCTTGTGGAAAGGACGAAACACCGTCCATTGACTGTAACAGCCCGTTTTAGAGCTAGAAATAGCAAGTT
Atxn3	ATCTTGTGGAAAGGACGAAACACCGACAGCAGCCTTCTGGAAATAGTTTTAGAGCTAGAAATAGCAAGTT
Atxn3	ATCTTGTGGAAAGGACGAAACACCGCTGTCATCCATATTTCCAGAGTTTTAGAGCTAGAAATAGCAAGTT
Atxn3	ATCTTGTGGAAAGGACGAAACACCGTAGGATCAATTCTGAGCCTCGTTTTAGAGCTAGAAATAGCAAGTT
Bap1	ATCTTGTGGAAAGGACGAAACACCGATCCTTCATTCGGCTCAGCGGTTTTAGAGCTAGAAATAGCAAGTT
Bap1	ATCTTGTGGAAAGGACGAAACACCGCCTGATCGTAGGTGTCAAAGGTTTTAGAGCTAGAAATAGCAAGTT
Bap1	ATCTTGTGGAAAGGACGAAACACCGTCTTACCGAAATCTTCCACCGTTTTAGAGCTAGAAATAGCAAGTT
Brcc3	GTGGAAAGGACGAAACACCGGCCGCAAGATGATGACAGAAGTTTTAGAGCTAGAAATAGC
Brcc3	GTGGAAAGGACGAAACACCGGTCCCATGAGAGTTGTTGGCGTTTTAGAGCTAGAAATAGC
Brcc3	GTGGAAAGGACGAAACACCGTACAGGTTGGCTGAACTAACGTTTTAGAGCTAGAAATAGC
Cops5	GTGGAAAGGACGAAACACCGACCAACCGATTGCATTCTCAGTTTTAGAGCTAGAAATAGC
Cops5	GTGGAAAGGACGAAACACCGAGCTGCTGCGTATGAGTATAGTTTTAGAGCTAGAAATAGC
Cops5	GTGGAAAGGACGAAACACCGTACCTTTGGATATGTCCTAAGTTTTAGAGCTAGAAATAGC
Cops6	GTGGAAAGGACGAAACACCGAGACATCCACGTCCATAAGCGTTTTAGAGCTAGAAATAGC
Cops6	GTGGAAAGGACGAAACACCGTGAGCTGCTGTCCCACACCGGTTTTAGAGCTAGAAATAGC
Cops6	GTGGAAAGGACGAAACACCGTTAGTGATTGGGGCTCTGATGTTTTAGAGCTAGAAATAGC
Cyld	ATCTTGTGGAAAGGACGAAACACCGCATTTCTAGGATAACCCTATGTTTTAGAGCTAGAAATAGCAAGTT
Cyld	ATCTTGTGGAAAGGACGAAACACCGGCAGGTTGAACCTCCCCCTTGTTTTAGAGCTAGAAATAGCAAGTT
Cyld	ATCTTGTGGAAAGGACGAAACACCGTCTCTTTAGGAAGAAGGTCGGTTTTAGAGCTAGAAATAGCAAGTT
Fam105a	ATCTTGTGGAAAGGACGAAACACCGACATACCTTCCGCATCAGTCGTTTTAGAGCTAGAAATAGCAAGTT
Fam105a	ATCTTGTGGAAAGGACGAAACACCGCAGGCCCGAAACTGTACTGCGTTTTAGAGCTAGAAATAGCAAGTT
Fam105a	ATCTTGTGGAAAGGACGAAACACCGTGGATCCAGCAGTACAGTTTGTTTTAGAGCTAGAAATAGCAAGTT
Fam105b	ATCTTGTGGAAAGGACGAAACACCGGAATTGCTAATACATGAAAGGTTTTAGAGCTAGAAATAGCAAGTT
Fam105b	ATCTTGTGGAAAGGACGAAACACCGGTGATAACTACTGTGCACTGGTTTTAGAGCTAGAAATAGCAAGTT
Fam105b	ATCTTGTGGAAAGGACGAAACACCGTAGTCCATGATATCCATTTCGTTTTAGAGCTAGAAATAGCAAGTT
Fam188a	ATCTTGTGGAAAGGACGAAACACCGGACAGGTGCAATAACAGCACGTTTTAGAGCTAGAAATAGCAAGTT
Fam188a	ATCTTGTGGAAAGGACGAAACACCGGGACCCCCGAGGAAGCGGCTGTTTTAGAGCTAGAAATAGCAAGTT
Fam188a	ATCTTGTGGAAAGGACGAAACACCGTCTCCTCCGAGAAGTCGTCCGTTTTAGAGCTAGAAATAGCAAGTT
Fam188b	ATCTTGTGGAAAGGACGAAACACCGCGGACATCATGGTCAGAAGCGTTTTAGAGCTAGAAATAGCAAGTT
Fam188b	ATCTTGTGGAAAGGACGAAACACCGTCCGCAAACGCTCTCTGAGAGTTTTAGAGCTAGAAATAGCAAGTT
Fam188b	ATCTTGTGGAAAGGACGAAACACCGTCTCCGCAAAGTTCTGCATCGTTTTAGAGCTAGAAATAGCAAGTT
Fam63a	ATCTTGTGGAAAGGACGAAACACCGATCACTCACCAAGGTGTGTCGTTTTAGAGCTAGAAATAGCAAGTT
Fam63a	ATCTTGTGGAAAGGACGAAACACCGCCTTCAACTTAACTTTCAGCGTTTTAGAGCTAGAAATAGCAAGTT
Fam63a	ATCTTGTGGAAAGGACGAAACACCGCGGGGTCCGTTCTCCCTTCCGTTTTAGAGCTAGAAATAGCAAGTT
Fam63b	ATCTTGTGGAAAGGACGAAACACCGCATAACTGCGGAGCAGCTGAGTTTTAGAGCTAGAAATAGCAAGTT
Fam63b	ATCTTGTGGAAAGGACGAAACACCGGACGTGAATGTCAGGTTCACGTTTTAGAGCTAGAAATAGCAAGTT
Fam63b	ATCTTGTGGAAAGGACGAAACACCGGGTGAAACTTCCACCAATGAGTTTTAGAGCTAGAAATAGCAAGTT
Josd1	ATCTTGTGGAAAGGACGAAACACCGAAAACATGAGTTGCGTGCCAGTTTTAGAGCTAGAAATAGCAAGTT
Josd1	ATCTTGTGGAAAGGACGAAACACCGCATTGCTCTTACTAATGTCAGTTTTAGAGCTAGAAATAGCAAGTT
Josd1	ATCTTGTGGAAAGGACGAAACACCGCCAAACAGCTTCATAGCCTTGTTTTAGAGCTAGAAATAGCAAGTT

Josd2	ATCTTGTGGAAAGGACGAAACACCGACCCAGTGTCGGCGGCGCAGGTTTTAGAGCTAGAAATAGCAAGTT
Josd2	ATCTTGTGGAAAGGACGAAACACCGGAACCCCCATCGCAGTCTCTGTTTTAGAGCTAGAAATAGCAAGTT
Josd2	ATCTTGTGGAAAGGACGAAACACCGTGACTCCATCCTCGTCCCCCGTTTTAGAGCTAGAAATAGCAAGTT
Mpnd	GTGGAAAGGACGAAACACCGACCTCATCGGCCGTAGCTACGTTTTAGAGCTAGAAATAGC
Mpnd	GTGGAAAGGACGAAACACCGACCTTGTCCATACGCTTCCCGTTTTAGAGCTAGAAATAGC
Mpnd	GTGGAAAGGACGAAACACCGTAACTGACCTGGCGGAGTCTGTTTTAGAGCTAGAAATAGC
Mysm1	GTGGAAAGGACGAAACACCGAGCTCTGTTCTCATCACTGAGTTTTAGAGCTAGAAATAGC
Mysm1	GTGGAAAGGACGAAACACCGATAATAAAAAATACACAAACGTTTTAGAGCTAGAAATAGC
Mysm1	GTGGAAAGGACGAAACACCGTTATCTAATAAATCACTTCCGTTTTAGAGCTAGAAATAGC
Otub1	ATCTTGTGGAAAGGACGAAACACCGACCTCACCTCTTGCTGAATTGTTTTAGAGCTAGAAATAGCAAGTT
Otub1	ATCTTGTGGAAAGGACGAAACACCGCCTCAGCATACTCCTTGTACGTTTTAGAGCTAGAAATAGCAAGTT
Otub1	ATCTTGTGGAAAGGACGAAACACCGGATTGCTGTGCAGAATCCTCGTTTTAGAGCTAGAAATAGCAAGTT
Otub2	ATCTTGTGGAAAGGACGAAACACCGACAGACCCCAAATGACCTTCGTTTTAGAGCTAGAAATAGCAAGTT
Otub2	ATCTTGTGGAAAGGACGAAACACCGGAGACGGAAACTGCTTCTACGTTTTAGAGCTAGAAATAGCAAGTT
Otub2	ATCTTGTGGAAAGGACGAAACACCGTTCAGGATGATCCCGAAGAAGTTTTAGAGCTAGAAATAGCAAGTT
Otud1	ATCTTGTGGAAAGGACGAAACACCGACCCGGTTCCGGCACCCGCTGTTTTAGAGCTAGAAATAGCAAGTT
Otud1	ATCTTGTGGAAAGGACGAAACACCGGCACATCGCCGTGCGGGCCCGTTTTAGAGCTAGAAATAGCAAGTT
Otud1	ATCTTGTGGAAAGGACGAAACACCGGGCCGGATATCCCGAATTGCGTTTTAGAGCTAGAAATAGCAAGTT
Otud3	ATCTTGTGGAAAGGACGAAACACCGATCAGCTTAATGCCCCTTTGGTTTTAGAGCTAGAAATAGCAAGTT
Otud3	ATCTTGTGGAAAGGACGAAACACCGGGATCACCACATTCAATTGAGTTTTAGAGCTAGAAATAGCAAGTT
Otud3	ATCTTGTGGAAAGGACGAAACACCGTCCGAGAGTGGCCCTCCAACGTTTTAGAGCTAGAAATAGCAAGTT
Otud4	ATCTTGTGGAAAGGACGAAACACCGAGGTGTTGCACTCTCAGTCTGTTTTAGAGCTAGAAATAGCAAGTT
Otud4	ATCTTGTGGAAAGGACGAAACACCGTGAAGAATATTTAAAACGTTGTTTTAGAGCTAGAAATAGCAAGTT
Otud4	ATCTTGTGGAAAGGACGAAACACCGTTTACCTGTACATAAGTGAAGTTTTAGAGCTAGAAATAGCAAGTT
Otud5	ATCTTGTGGAAAGGACGAAACACCGACCTACAGCCCGAAATAGACGTTTTAGAGCTAGAAATAGCAAGTT
Otud5	ATCTTGTGGAAAGGACGAAACACCGAGAGATGTACAACCGTCCTGGTTTTAGAGCTAGAAATAGCAAGTT
Otud5	ATCTTGTGGAAAGGACGAAACACCGAGGACGGTGCCTGTCTATTTGTTTTAGAGCTAGAAATAGCAAGTT
Otud6a	ATCTTGTGGAAAGGACGAAACACCGACTGGCTGCCAACCGTCGTGGTTTTAGAGCTAGAAATAGCAAGTT
Otud6a	ATCTTGTGGAAAGGACGAAACACCGCGTCTGCCCTTGTCGTTCTTGTTTTAGAGCTAGAAATAGCAAGTT
Otud6a	ATCTTGTGGAAAGGACGAAACACCGGCGCGACCGAAGAGCTCACCGTTTTAGAGCTAGAAATAGCAAGTT
Otud6b	ATCTTGTGGAAAGGACGAAACACCGCACGGAAGATGTTGCTAAATGTTTTAGAGCTAGAAATAGCAAGTT
Otud6b	ATCTTGTGGAAAGGACGAAACACCGTGCTGTTAACATTTCAAACTGTTTTAGAGCTAGAAATAGCAAGTT
Otud6b	ATCTTGTGGAAAGGACGAAACACCGTTCCCAAAAACGACAAAAAGGTTTTAGAGCTAGAAATAGCAAGTT
Otud7a	ATCTTGTGGAAAGGACGAAACACCGAGTCCTCACTGTACACGCTCGTTTTAGAGCTAGAAATAGCAAGTT
Otud7a	ATCTTGTGGAAAGGACGAAACACCGAGTCGCTTAGTGCAGCTGTCGTTTTAGAGCTAGAAATAGCAAGTT
Otud7a	ATCTTGTGGAAAGGACGAAACACCGCCTTTCCAGGTCGCCTGAACGTTTTAGAGCTAGAAATAGCAAGTT
Otud7b	ATCTTGTGGAAAGGACGAAACACCGCCATCCCCAGTAGTTGCTAAGTTTTAGAGCTAGAAATAGCAAGTT
Otud7b	ATCTTGTGGAAAGGACGAAACACCGGGGTTTCCATGATAGAGACTGTTTTAGAGCTAGAAATAGCAAGTT
Otud7b	ATCTTGTGGAAAGGACGAAACACCGTGTCTGCAGGGCGTCTGAATGTTTTAGAGCTAGAAATAGCAAGTT
Prpf8	GTGGAAAGGACGAAACACCGACGTCCCGGATCTGCTCCCAGTTTTAGAGCTAGAAATAGC
Prpf8	GTGGAAAGGACGAAACACCGCCTTTGTTAACGAGATTCCCGTTTTAGAGCTAGAAATAGC
Prpf8	GTGGAAAGGACGAAACACCGGATGTCCACACTCTACCGACGTTTTAGAGCTAGAAATAGC
Stambp	GTGGAAAGGACGAAACACCGCGTGGGCGAGAACGGTGAGCGTTTTAGAGCTAGAAATAGC

Stambp	GTGGAAAGGACGAAACACCGGATGTGGCGCAGGCCTTCAAGTTTTAGAGCTAGAAATAGC
Stambp	GTGGAAAGGACGAAACACCGTGAGCAGTATAAAGAGCGAAGTTTTAGAGCTAGAAATAGC
Stambpl1	GTGGAAAGGACGAAACACCGAATTGAAAACGGACCTGCTAGTTTTAGAGCTAGAAATAGC
Stambpl1	GTGGAAAGGACGAAACACCGCTGCACACTGCTGGTAATCTGTTTTAGAGCTAGAAATAGC
Stambpl1	GTGGAAAGGACGAAACACCGGTTCAGACTCTAGTTGCTGCGTTTTAGAGCTAGAAATAGC
Tnfaip3	ATCTTGTGGAAAGGACGAAACACCGCAGGACTTTGCTACGACACTGTTTTAGAGCTAGAAATAGCAAGTT
Tnfaip3	ATCTTGTGGAAAGGACGAAACACCGCTCGGAACTTTAAATTCCGCGTTTTAGAGCTAGAAATAGCAAGTT
Tnfaip3	ATCTTGTGGAAAGGACGAAACACCGCTTACTTTGCAGCGTTGATCGTTTTAGAGCTAGAAATAGCAAGTT
Uchl1	ATCTTGTGGAAAGGACGAAACACCGCAAGTGTTTCGAGAAGAACGGTTTTAGAGCTAGAAATAGCAAGTT
Uchl1	ATCTTGTGGAAAGGACGAAACACCGCTCGTTCTTCTCGAAACACTGTTTTAGAGCTAGAAATAGCAAGTT
Uchl1	ATCTTGTGGAAAGGACGAAACACCGCTGGCCGGCGACCCCCAGCTGTTTTAGAGCTAGAAATAGCAAGTT
Uchl3	ATCTTGTGGAAAGGACGAAACACCGAACAAAGACAAGATGCACTTGTTTTAGAGCTAGAAATAGCAAGTT
Uchl3	ATCTTGTGGAAAGGACGAAACACCGGGTACCATGCTAAGAAGTTCGTTTTAGAGCTAGAAATAGCAAGTT
Uchl3	ATCTTGTGGAAAGGACGAAACACCGTACTGACGTCATAGTTCTCCGTTTTAGAGCTAGAAATAGCAAGTT
Uchl5	ATCTTGTGGAAAGGACGAAACACCGAGAAAAATTAACCCATGAACGTTTTAGAGCTAGAAATAGCAAGTT
Uchl5	ATCTTGTGGAAAGGACGAAACACCGAGAAGAACCTGCTGGCTCTGGTTTTAGAGCTAGAAATAGCAAGTT
Uchl5	ATCTTGTGGAAAGGACGAAACACCGAGCTCCATATTTCTTCTACTGTTTTAGAGCTAGAAATAGCAAGTT
Usp1	ATCTTGTGGAAAGGACGAAACACCGGAAGGATGACTCTAATCAGAGTTTTAGAGCTAGAAATAGCAAGTT
Usp1	ATCTTGTGGAAAGGACGAAACACCGTCAGATAACAAGTGTTGCCAGTTTTAGAGCTAGAAATAGCAAGTT
Usp1	ATCTTGTGGAAAGGACGAAACACCGTTCACTCCAGTCTTAAAGCCGTTTTAGAGCTAGAAATAGCAAGTT
Usp10	ATCTTGTGGAAAGGACGAAACACCGATGACACTGGCTTATGTATTGTTTTAGAGCTAGAAATAGCAAGTT
Usp10	ATCTTGTGGAAAGGACGAAACACCGCCAGTGTCATTGCAACCCCGGTTTTAGAGCTAGAAATAGCAAGTT
Usp10	ATCTTGTGGAAAGGACGAAACACCGGACTCCCCGCTCTTCTGTTGGTTTTAGAGCTAGAAATAGCAAGTT
Usp11	ATCTTGTGGAAAGGACGAAACACCGACAGTGGGAGGTGTACGTGAGTTTTAGAGCTAGAAATAGCAAGTT
Usp11	ATCTTGTGGAAAGGACGAAACACCGCCAGCATTGTTGATACAGCCGTTTTAGAGCTAGAAATAGCAAGTT
Usp11	ATCTTGTGGAAAGGACGAAACACCGTCACAGGTAATAGAACTTCCGTTTTAGAGCTAGAAATAGCAAGTT
Usp12	ATCTTGTGGAAAGGACGAAACACCGACAGCACACCAGACCCAACCGTTTTAGAGCTAGAAATAGCAAGTT
Usp12	ATCTTGTGGAAAGGACGAAACACCGCATCAATTACTCACTGCTTAGTTTTAGAGCTAGAAATAGCAAGTT
Usp12	ATCTTGTGGAAAGGACGAAACACCGCGCCCAAGAAGTTCATCACAGTTTTAGAGCTAGAAATAGCAAGTT
Usp13	ATCTTGTGGAAAGGACGAAACACCGGAAAGACACTTTCGAAAAACGTTTTAGAGCTAGAAATAGCAAGTT
Usp13	ATCTTGTGGAAAGGACGAAACACCGTGCCAACAACCTTGTGCAACGTTTTAGAGCTAGAAATAGCAAGTT
Usp13	ATCTTGTGGAAAGGACGAAACACCGTTTGGGTAAAGCTCCACCAGGTTTTAGAGCTAGAAATAGCAAGTT
Usp14	ATCTTGTGGAAAGGACGAAACACCGCTTTGAAATAGGATGATGACGTTTTAGAGCTAGAAATAGCAAGTT
Usp14	ATCTTGTGGAAAGGACGAAACACCGGCCAGCCAGACAAAAAGTTAGTTTTAGAGCTAGAAATAGCAAGTT
Usp14	ATCTTGTGGAAAGGACGAAACACCGTCATGTAACAAGTGTTACCAGTTTTAGAGCTAGAAATAGCAAGTT
Usp15	ATCTTGTGGAAAGGACGAAACACCGCATATACTTGTTCCACAGTCGTTTTAGAGCTAGAAATAGCAAGTT
Usp15	ATCTTGTGGAAAGGACGAAACACCGCCTGGACCCATCGATAACTCGTTTTAGAGCTAGAAATAGCAAGTT
Usp15	ATCTTGTGGAAAGGACGAAACACCGGCTGGAATAAACTTGTCAGCGTTTTAGAGCTAGAAATAGCAAGTT
Usp16	ATCTTGTGGAAAGGACGAAACACCGAAGCAGAAGACCCTTCGGTTGTTTTAGAGCTAGAAATAGCAAGTT
Usp16	ATCTTGTGGAAAGGACGAAACACCGGTCTGGACAACTGGAGCGTCGTTTTAGAGCTAGAAATAGCAAGTT
Usp16	ATCTTGTGGAAAGGACGAAACACCGTTAAGACAGAGCCAAACCGAGTTTTAGAGCTAGAAATAGCAAGTT
Usp17la	ATCTTGTGGAAAGGACGAAACACCGGATGGTGCGACTTCTCACAGGTTTTAGAGCTAGAAATAGCAAGTT
Usp17la	ATCTTGTGGAAAGGACGAAACACCGGTTGCTCTTTCCTTCCCAGAGTTTTAGAGCTAGAAATAGCAAGTT

Usp17la	ATCTTGTGGAAAGGACGAAACACCGTCTCCTAAATCTTCTGTGAAGTTTTAGAGCTAGAAATAGCAAGTT
Usp17lb	ATCTTGTGGAAAGGACGAAACACCGACTATAAAGCTTTAGGGTCTGTTTTAGAGCTAGAAATAGCAAGTT
Usp17lb	ATCTTGTGGAAAGGACGAAACACCGATGAGACTTACCTTCTGGGAGTTTTAGAGCTAGAAATAGCAAGTT
Usp17lb	ATCTTGTGGAAAGGACGAAACACCGTCGACTCAGTGGTGCAATTGGTTTTAGAGCTAGAAATAGCAAGTT
Usp17lc	ATCTTGTGGAAAGGACGAAACACCGGAATACCAAACTTGTGCCTCGTTTTAGAGCTAGAAATAGCAAGTT
Usp17lc	ATCTTGTGGAAAGGACGAAACACCGGTTTCTCTTTCCTTCCCAGAGTTTTAGAGCTAGAAATAGCAAGTT
Usp17lc	ATCTTGTGGAAAGGACGAAACACCGTGTGTTTCTGCCCAAGTTTCGTTTTAGAGCTAGAAATAGCAAGTT
Usp17ld	ATCTTGTGGAAAGGACGAAACACCGGTGAGACTTACCTTCTGGGAGTTTTAGAGCTAGAAATAGCAAGTT
Usp17ld	ATCTTGTGGAAAGGACGAAACACCGTGTCAATACTGACCTCTTTGGTTTTAGAGCTAGAAATAGCAAGTT
Usp17ld	ATCTTGTGGAAAGGACGAAACACCGTGTGTTTCTGCCCCAGTTTCGTTTTAGAGCTAGAAATAGCAAGTT
Usp17le	ATCTTGTGGAAAGGACGAAACACCGCCTTACTTGCAGAGACTGGAGTTTTAGAGCTAGAAATAGCAAGTT
Usp17le	ATCTTGTGGAAAGGACGAAACACCGGTGAGACTTACCTTCTGGGAGTTTTAGAGCTAGAAATAGCAAGTT
Usp17le	ATCTTGTGGAAAGGACGAAACACCGGTTTCTCTTTCCTTCCCAGAGTTTTAGAGCTAGAAATAGCAAGTT
Usp18	ATCTTGTGGAAAGGACGAAACACCGCTGGTTGGTTTACACAACATGTTTTAGAGCTAGAAATAGCAAGTT
Usp18	ATCTTGTGGAAAGGACGAAACACCGCTTGCAGACAGAGCGGCTGCGTTTTAGAGCTAGAAATAGCAAGTT
Usp18	ATCTTGTGGAAAGGACGAAACACCGTAGTCAGGTTCCAGATTGTAGTTTTAGAGCTAGAAATAGCAAGTT
Usp19	ATCTTGTGGAAAGGACGAAACACCGAAGTTCTTGCACCAAAGTGCGTTTTAGAGCTAGAAATAGCAAGTT
Usp19	ATCTTGTGGAAAGGACGAAACACCGACCCTTGCGAGCCTGCACTTGTTTTAGAGCTAGAAATAGCAAGTT
Usp19	ATCTTGTGGAAAGGACGAAACACCGAGCGGCTCAAGATGTCTGCAGTTTTAGAGCTAGAAATAGCAAGTT
Usp2	ATCTTGTGGAAAGGACGAAACACCGCTGGCTGGTCTTCGAAACCTGTTTTAGAGCTAGAAATAGCAAGTT
Usp2	ATCTTGTGGAAAGGACGAAACACCGGGTCCCGCATGTACAGCCTCGTTTTAGAGCTAGAAATAGCAAGTT
Usp2	ATCTTGTGGAAAGGACGAAACACCGTTACTTACGGGAGATGATCAGTTTTAGAGCTAGAAATAGCAAGTT
Usp20	ATCTTGTGGAAAGGACGAAACACCGCAGAAAGACTTCACGCTCACGTTTTAGAGCTAGAAATAGCAAGTT
Usp20	ATCTTGTGGAAAGGACGAAACACCGCAGGTCACCTGCCCTTACGTGTTTTAGAGCTAGAAATAGCAAGTT
Usp20	ATCTTGTGGAAAGGACGAAACACCGGACTCTCCACAGCCAACGTAGTTTTAGAGCTAGAAATAGCAAGTT
Usp21	ATCTTGTGGAAAGGACGAAACACCGCATGTTGGCCTCCGAAATCTGTTTTAGAGCTAGAAATAGCAAGTT
Usp21	ATCTTGTGGAAAGGACGAAACACCGCGCAGACAGAAGTCTCGAAGGTTTTAGAGCTAGAAATAGCAAGTT
Usp21	ATCTTGTGGAAAGGACGAAACACCGTTCGAGACTTCTGTCTGCGAGTTTTAGAGCTAGAAATAGCAAGTT
Usp22	ATCTTGTGGAAAGGACGAAACACCGAGTAAATACCTCCGTACATCGTTTTAGAGCTAGAAATAGCAAGTT
Usp22	ATCTTGTGGAAAGGACGAAACACCGCAAGTCAAAGCGACACAACCGTTTTAGAGCTAGAAATAGCAAGTT
Usp22	ATCTTGTGGAAAGGACGAAACACCGTCACCTACCTATGGTACAATGTTTTAGAGCTAGAAATAGCAAGTT
Usp24	ATCTTGTGGAAAGGACGAAACACCGAAACAGGATCCAATTCCCATGTTTTAGAGCTAGAAATAGCAAGTT
Usp24	ATCTTGTGGAAAGGACGAAACACCGCCTTACCTTCTTAAATGCCTGTTTTAGAGCTAGAAATAGCAAGTT
Usp24	ATCTTGTGGAAAGGACGAAACACCGGTTGATGCTACTAATAGAATGTTTTAGAGCTAGAAATAGCAAGTT
Usp25	ATCTTGTGGAAAGGACGAAACACCGGAATCCCTCCAAACTTCTATGTTTTAGAGCTAGAAATAGCAAGTT
Usp25	ATCTTGTGGAAAGGACGAAACACCGGGGCATAACCGATGAAGAGCGTTTTAGAGCTAGAAATAGCAAGTT
Usp25	ATCTTGTGGAAAGGACGAAACACCGGTTTGACTCCGCCAAACTCAGTTTTAGAGCTAGAAATAGCAAGTT
Usp26	ATCTTGTGGAAAGGACGAAACACCGGGAGGCCCAAAAGTACCAACGTTTTAGAGCTAGAAATAGCAAGTT
Usp26	ATCTTGTGGAAAGGACGAAACACCGGGTCTTCGCCATAGGTTTGAGTTTTAGAGCTAGAAATAGCAAGTT
Usp26	ATCTTGTGGAAAGGACGAAACACCGGTTTATAGTTTCGAAACCCTGTTTTAGAGCTAGAAATAGCAAGTT
Usp27x	ATCTTGTGGAAAGGACGAAACACCGAATTAGTCCTCGTAAGCCGAGTTTTAGAGCTAGAAATAGCAAGTT
Usp27x	ATCTTGTGGAAAGGACGAAACACCGACAAGCGTCCACCTCGACAGGTTTTAGAGCTAGAAATAGCAAGTT
Usp27x	ATCTTGTGGAAAGGACGAAACACCGGACGAGCGTGTATCCACACCGTTTTAGAGCTAGAAATAGCAAGTT

Usp28	ATCTTGTGGAAAGGACGAAACACCGAATCAGCTGCGAGAAATCACGTTTTAGAGCTAGAAATAGCAAGTT
Usp28	ATCTTGTGGAAAGGACGAAACACCGATCCACTCTCCTCCAGTTATGTTTTAGAGCTAGAAATAGCAAGTT
Usp28	ATCTTGTGGAAAGGACGAAACACCGCGACAACAAAGATGACCTGCGTTTTAGAGCTAGAAATAGCAAGTT
Usp29	ATCTTGTGGAAAGGACGAAACACCGCATACACATCACTTACGTAAGTTTTAGAGCTAGAAATAGCAAGTT
Usp29	ATCTTGTGGAAAGGACGAAACACCGCCTTCTTAGATAACATTGACGTTTTAGAGCTAGAAATAGCAAGTT
Usp29	ATCTTGTGGAAAGGACGAAACACCGCGTAGTGGTTAGTGGTGAGCGTTTTAGAGCTAGAAATAGCAAGTT
Usp3	ATCTTGTGGAAAGGACGAAACACCGACTTGTTCAAGTGTCCACTGGTTTTAGAGCTAGAAATAGCAAGTT
Usp3	ATCTTGTGGAAAGGACGAAACACCGCAATAGCTCAGCCTTTACAGGTTTTAGAGCTAGAAATAGCAAGTT
Usp3	ATCTTGTGGAAAGGACGAAACACCGGCAGTAGCTACAGCACATACGTTTTAGAGCTAGAAATAGCAAGTT
Usp30	ATCTTGTGGAAAGGACGAAACACCGCAGCAGGGATCTATGTTATTGTTTTAGAGCTAGAAATAGCAAGTT
Usp30	ATCTTGTGGAAAGGACGAAACACCGCCTGCCCTGCGTTTGTCAAGGTTTTAGAGCTAGAAATAGCAAGTT
Usp30	ATCTTGTGGAAAGGACGAAACACCGCTTGTTTGATGTGCATTCCCGTTTTAGAGCTAGAAATAGCAAGTT
Usp31	ATCTTGTGGAAAGGACGAAACACCGAAGAATGCGTTGCAGTATCGGTTTTAGAGCTAGAAATAGCAAGTT
Usp31	ATCTTGTGGAAAGGACGAAACACCGGGCATCATATCTGTCTCTGAGTTTTAGAGCTAGAAATAGCAAGTT
Usp31	ATCTTGTGGAAAGGACGAAACACCGTCTTACCTTGTATGGGGTAGGTTTTAGAGCTAGAAATAGCAAGTT
Usp32	ATCTTGTGGAAAGGACGAAACACCGCATTTCAATAACTTAATAGTGTTTTAGAGCTAGAAATAGCAAGTT
Usp32	ATCTTGTGGAAAGGACGAAACACCGGTTATTGAAATGCAGCCCTTGTTTTAGAGCTAGAAATAGCAAGTT
Usp32	ATCTTGTGGAAAGGACGAAACACCGTCCTACTTTCTACCAAACTCGTTTTAGAGCTAGAAATAGCAAGTT
Usp33	ATCTTGTGGAAAGGACGAAACACCGAGTTGGTGAAATAACGAAAGGTTTTAGAGCTAGAAATAGCAAGTT
Usp33	ATCTTGTGGAAAGGACGAAACACCGATACCTCCAGGCACGCCCACGTTTTAGAGCTAGAAATAGCAAGTT
Usp33	ATCTTGTGGAAAGGACGAAACACCGCGACTCCCCGCAGCCAACATGTTTTAGAGCTAGAAATAGCAAGTT
Usp34	ATCTTGTGGAAAGGACGAAACACCGAACTGCATACCTCTGAGTCCGTTTTAGAGCTAGAAATAGCAAGTT
Usp34	ATCTTGTGGAAAGGACGAAACACCGAGCAATCCTTCATGAGAGAAGTTTTAGAGCTAGAAATAGCAAGTT
Usp34	ATCTTGTGGAAAGGACGAAACACCGGACATAACGAAGCAAATGCAGTTTTAGAGCTAGAAATAGCAAGTT
Usp35	ATCTTGTGGAAAGGACGAAACACCGAGCTGCTGTACCCCATCGTGGTTTTAGAGCTAGAAATAGCAAGTT
Usp35	ATCTTGTGGAAAGGACGAAACACCGCGTGGTCCAGCACCTCCCATGTTTTAGAGCTAGAAATAGCAAGTT
Usp35	ATCTTGTGGAAAGGACGAAACACCGGGAGCCACCGTCCAGCGCCTGTTTTAGAGCTAGAAATAGCAAGTT
Usp36	ATCTTGTGGAAAGGACGAAACACCGACAGCTCCGTGCGTGCTCCTGTTTTAGAGCTAGAAATAGCAAGTT
Usp36	ATCTTGTGGAAAGGACGAAACACCGTAGGTTGGATCGGCAGACACGTTTTAGAGCTAGAAATAGCAAGTT
Usp36	ATCTTGTGGAAAGGACGAAACACCGTCGCATCAATGGTGTACCGCGTTTTAGAGCTAGAAATAGCAAGTT
Usp37	ATCTTGTGGAAAGGACGAAACACCGCCAGCTATGAAAGCTTCTCAGTTTTAGAGCTAGAAATAGCAAGTT
Usp37	ATCTTGTGGAAAGGACGAAACACCGGCTTTCTTACTCAGACAATCGTTTTAGAGCTAGAAATAGCAAGTT
Usp37	ATCTTGTGGAAAGGACGAAACACCGTATTGACAAAGTACCAAGTAGTTTTAGAGCTAGAAATAGCAAGTT
Usp38	ATCTTGTGGAAAGGACGAAACACCGCAAGGCAACCGAGGGCTCAAGTTTTAGAGCTAGAAATAGCAAGTT
Usp38	ATCTTGTGGAAAGGACGAAACACCGCTTACCACATGGAATGCCTCGTTTTAGAGCTAGAAATAGCAAGTT
Usp38	ATCTTGTGGAAAGGACGAAACACCGTGGCTCAGCATGTAGATACGGTTTTAGAGCTAGAAATAGCAAGTT
Usp39	ATCTTGTGGAAAGGACGAAACACCGAAGTGGTGCCATCATATGCCGTTTTAGAGCTAGAAATAGCAAGTT
Usp39	ATCTTGTGGAAAGGACGAAACACCGCACAGCATTTGCATAGTCGTGTTTTAGAGCTAGAAATAGCAAGTT
Usp39	ATCTTGTGGAAAGGACGAAACACCGTGAAATCATTGATTCCTCGCGTTTTAGAGCTAGAAATAGCAAGTT
Usp4	ATCTTGTGGAAAGGACGAAACACCGCCTGGACCTATTGACAACTCGTTTTAGAGCTAGAAATAGCAAGTT
Usp4	ATCTTGTGGAAAGGACGAAACACCGCGATGAGCTGGACTATGTGCGTTTTAGAGCTAGAAATAGCAAGTT
Usp4	ATCTTGTGGAAAGGACGAAACACCGTAGCTTGCTCAACTGCTCATGTTTTAGAGCTAGAAATAGCAAGTT
Usp40	ATCTTGTGGAAAGGACGAAACACCGCACAGGTTCCACAGTGGTACGTTTTAGAGCTAGAAATAGCAAGTT

Usp40	ATCTTGTGGAAAGGACGAAACACCGCCTTTGCTGCTTTAACCAGTGTTTTAGAGCTAGAAATAGCAAGTT
Usp40	ATCTTGTGGAAAGGACGAAACACCGTACACTCTTTGCAAACAATCGTTTTAGAGCTAGAAATAGCAAGTT
Usp42	ATCTTGTGGAAAGGACGAAACACCGATAGCTAGACACTTCCGTTTGTTTTAGAGCTAGAAATAGCAAGTT
Usp42	ATCTTGTGGAAAGGACGAAACACCGTATCTTGCAGGCCACGCAGAGTTTTAGAGCTAGAAATAGCAAGTT
Usp42	ATCTTGTGGAAAGGACGAAACACCGTCATACAAAATCCTTCTGCGGTTTTAGAGCTAGAAATAGCAAGTT
Usp43	ATCTTGTGGAAAGGACGAAACACCGCAATTCCCAGCACGATGCCCGTTTTAGAGCTAGAAATAGCAAGTT
Usp43	ATCTTGTGGAAAGGACGAAACACCGGCCACTTTCAAGCACAGTACGTTTTAGAGCTAGAAATAGCAAGTT
Usp43	ATCTTGTGGAAAGGACGAAACACCGGCTTCTGGATCGTGTCCACGGTTTTAGAGCTAGAAATAGCAAGTT
Usp44	ATCTTGTGGAAAGGACGAAACACCGGTACATGTCGTTCACCTCGAGTTTTAGAGCTAGAAATAGCAAGTT
Usp44	ATCTTGTGGAAAGGACGAAACACCGGTTCTAAACGATAACGCAGCGTTTTAGAGCTAGAAATAGCAAGTT
Usp44	ATCTTGTGGAAAGGACGAAACACCGTATTCTGTATGGATCGAGCGGTTTTAGAGCTAGAAATAGCAAGTT
Usp45	ATCTTGTGGAAAGGACGAAACACCGACATTGCAACAAGAAAGTTTGTTTTAGAGCTAGAAATAGCAAGTT
Usp45	ATCTTGTGGAAAGGACGAAACACCGACCATATGACCCATGTACTCGTTTTAGAGCTAGAAATAGCAAGTT
Usp45	ATCTTGTGGAAAGGACGAAACACCGTTGTCATCAGCCTGAGTACAGTTTTAGAGCTAGAAATAGCAAGTT
Usp46	ATCTTGTGGAAAGGACGAAACACCGGAAGTACAGCGCTTGAAGCAGTTTTAGAGCTAGAAATAGCAAGTT
Usp46	ATCTTGTGGAAAGGACGAAACACCGGGAGAATGTGTTGGCATACAGTTTTAGAGCTAGAAATAGCAAGTT
Usp46	ATCTTGTGGAAAGGACGAAACACCGTCTTGACCTCTCTGTCGATGGTTTTAGAGCTAGAAATAGCAAGTT
Usp47	ATCTTGTGGAAAGGACGAAACACCGATTTATGTAGCCTACTTTGTGTTTTAGAGCTAGAAATAGCAAGTT
Usp47	ATCTTGTGGAAAGGACGAAACACCGTTAGGAGGACTCCAATAACGGTTTTAGAGCTAGAAATAGCAAGTT
Usp47	ATCTTGTGGAAAGGACGAAACACCGTTATGTGGGATTAGTAAACCGTTTTAGAGCTAGAAATAGCAAGTT
Usp48	ATCTTGTGGAAAGGACGAAACACCGCCAATGCCAACCAAGCAATTGTTTTAGAGCTAGAAATAGCAAGTT
Usp48	ATCTTGTGGAAAGGACGAAACACCGCCGAATTGCTTGGTTGGCATGTTTTAGAGCTAGAAATAGCAAGTT
Usp48	ATCTTGTGGAAAGGACGAAACACCGTGCACAACATTCCGAACATCGTTTTAGAGCTAGAAATAGCAAGTT
Usp49	ATCTTGTGGAAAGGACGAAACACCGAATGATCTACAGACTACCTCGTTTTAGAGCTAGAAATAGCAAGTT
Usp49	ATCTTGTGGAAAGGACGAAACACCGCCAATACCATTGAGCCCTTTGTTTTAGAGCTAGAAATAGCAAGTT
Usp49	ATCTTGTGGAAAGGACGAAACACCGGGCTACACCTTAAAAGATTCGTTTTAGAGCTAGAAATAGCAAGTT
Usp5	ATCTTGTGGAAAGGACGAAACACCGAGATCGCTCGGGATGGGTTGGTTTTAGAGCTAGAAATAGCAAGTT
Usp5	ATCTTGTGGAAAGGACGAAACACCGCACCTCGGAAGAAGCCCACCGTTTTAGAGCTAGAAATAGCAAGTT
Usp5	ATCTTGTGGAAAGGACGAAACACCGTTGGGATGGGGAAGTCCGTCGTTTTAGAGCTAGAAATAGCAAGTT
Usp50	ATCTTGTGGAAAGGACGAAACACCGACAATAGATTTGGTTGCTCCGTTTTAGAGCTAGAAATAGCAAGTT
Usp50	ATCTTGTGGAAAGGACGAAACACCGCCTACCTGATGACAGATATGGTTTTAGAGCTAGAAATAGCAAGTT
Usp50	ATCTTGTGGAAAGGACGAAACACCGTGTGAGGACCACGATTTCCAGTTTTAGAGCTAGAAATAGCAAGTT
Usp51	ATCTTGTGGAAAGGACGAAACACCGCTAGTAGTAGCACATCGAGTGTTTTAGAGCTAGAAATAGCAAGTT
Usp51	ATCTTGTGGAAAGGACGAAACACCGGCTGGACATGACCCCGTTCTGTTTTAGAGCTAGAAATAGCAAGTT
Usp51	ATCTTGTGGAAAGGACGAAACACCGGGGTCTAATAAGGGTCCCACGTTTTAGAGCTAGAAATAGCAAGTT
Usp53	ATCTTGTGGAAAGGACGAAACACCGATGAGTCACACAAGACTTAGGTTTTAGAGCTAGAAATAGCAAGTT
Usp53	ATCTTGTGGAAAGGACGAAACACCGTCAGAGCTCTCAAGCTTCTTGTTTTAGAGCTAGAAATAGCAAGTT
Usp53	ATCTTGTGGAAAGGACGAAACACCGTGCCATGACTCTGTATGAGCGTTTTAGAGCTAGAAATAGCAAGTT
Usp54	ATCTTGTGGAAAGGACGAAACACCGAAAGGGCAGCGGGTCGGACGGTTTTAGAGCTAGAAATAGCAAGTT
Usp54	ATCTTGTGGAAAGGACGAAACACCGCCCGCTGCCCTTTATCCAGAGTTTTAGAGCTAGAAATAGCAAGTT
Usp54	ATCTTGTGGAAAGGACGAAACACCGTTGTAAGCTGCCGAAAGCTAGTTTTAGAGCTAGAAATAGCAAGTT
Usp6nl	ATCTTGTGGAAAGGACGAAACACCGAAATCGAAAGGACTTCCAAGGTTTTAGAGCTAGAAATAGCAAGTT
Usp6nl	ATCTTGTGGAAAGGACGAAACACCGCCCATACCATAATGCAGCTGGTTTTAGAGCTAGAAATAGCAAGTT

Usp6nl	ATCTTGTGGAAAGGACGAAACACCGTCTCTCCACAGATTCCGACCGTTTTAGAGCTAGAAATAGCAAGTT
Usp7	ATCTTGTGGAAAGGACGAAACACCGAGAACAAGTGGCTGATTCGCGTTTTAGAGCTAGAAATAGCAAGTT
Usp7	ATCTTGTGGAAAGGACGAAACACCGTCTTCAGCACTGCTTGTGCGGTTTTAGAGCTAGAAATAGCAAGTT
Usp7	ATCTTGTGGAAAGGACGAAACACCGTGCCTGTACAAAAACTTCAAGTTTTAGAGCTAGAAATAGCAAGTT
Usp8	ATCTTGTGGAAAGGACGAAACACCGACCTGCTGTTGCTTGAAATCGTTTTAGAGCTAGAAATAGCAAGTT
Usp8	ATCTTGTGGAAAGGACGAAACACCGTCTCCGAAAGCCTTAAACTAGTTTTAGAGCTAGAAATAGCAAGTT
Usp8	ATCTTGTGGAAAGGACGAAACACCGTTGCAATACCCTTTGGGTTTGTTTTAGAGCTAGAAATAGCAAGTT
Usp9x	ATCTTGTGGAAAGGACGAAACACCGAAAACTGGAACAACCCATCGGTTTTAGAGCTAGAAATAGCAAGTT
Usp9x	ATCTTGTGGAAAGGACGAAACACCGAGTTAGATGATATGATCAACGTTTTAGAGCTAGAAATAGCAAGTT
Usp9x	ATCTTGTGGAAAGGACGAAACACCGGCTATTGATCTTAGTAAGAAGTTTTAGAGCTAGAAATAGCAAGTT
Usp9y	ATCTTGTGGAAAGGACGAAACACCGCACAAAGCTCCACCAGGCGAGTTTTAGAGCTAGAAATAGCAAGTT
Usp9y	ATCTTGTGGAAAGGACGAAACACCGTCACCTTGGCCTTGTTCATAGTTTTAGAGCTAGAAATAGCAAGTT
Usp9y	ATCTTGTGGAAAGGACGAAACACCGTTGTGTGGCCAAATTGTCTCGTTTTAGAGCTAGAAATAGCAAGTT
Uspl1	ATCTTGTGGAAAGGACGAAACACCGGGTTCAAGCTTTAACAGTACGTTTTAGAGCTAGAAATAGCAAGTT
Uspl1	ATCTTGTGGAAAGGACGAAACACCGTCAACACCTCCAAGTGCACCGTTTTAGAGCTAGAAATAGCAAGTT
Uspl1	ATCTTGTGGAAAGGACGAAACACCGTTACCAATATTGTTCCTGAGGTTTTAGAGCTAGAAATAGCAAGTT
Vcpip1	ATCTTGTGGAAAGGACGAAACACCGACTTCCTCACATGTTCCAGCGTTTTAGAGCTAGAAATAGCAAGTT
Vcpip1	ATCTTGTGGAAAGGACGAAACACCGGAGCTTATTCGAATAGCTCCGTTTTAGAGCTAGAAATAGCAAGTT
Vcpip1	ATCTTGTGGAAAGGACGAAACACCGTGTAGAATACACCACCCTGCGTTTTAGAGCTAGAAATAGCAAGTT
Yod1	GTGGAAAGGACGAAACACCGACCCGCCACATAGTGTCGGCGTTTTAGAGCTAGAAATAGC
Yod1	GTGGAAAGGACGAAACACCGTGCCGGTGATAGCGGCGATTGTTTTAGAGCTAGAAATAGC
Yod1	GTGGAAAGGACGAAACACCGTTCCACGACATAGTACACACGTTTTAGAGCTAGAAATAGC
Zranb1	ATCTTGTGGAAAGGACGAAACACCGATGACAAAGACTCGGTGCTTGTTTTAGAGCTAGAAATAGCAAGTT
Zranb1	ATCTTGTGGAAAGGACGAAACACCGCTGAACTGACAGAGCAAATCGTTTTAGAGCTAGAAATAGCAAGTT
Zranb1	ATCTTGTGGAAAGGACGAAACACCGGTAACATTTACATTACCAGCGTTTTAGAGCTAGAAATAGCAAGTT
Zranb1	GTGGAAAGGACGAAACACCGATGACAAAGACTCGGTGCTTGTTTTAGAGCTAGAAATAGC
Zranb1	GTGGAAAGGACGAAACACCGCTGAACTGACAGAGCAAATCGTTTTAGAGCTAGAAATAGC
Zranb1	GTGGAAAGGACGAAACACCGGTAACATTTACATTACCAGCGTTTTAGAGCTAGAAATAGC
Zufsp	ATCTTGTGGAAAGGACGAAACACCGATAGCTATTGGTATTGATGAGTTTTAGAGCTAGAAATAGCAAGTT
Zufsp	ATCTTGTGGAAAGGACGAAACACCGATTCTCTATCACTAGAACACGTTTTAGAGCTAGAAATAGCAAGTT
Zufsp	ATCTTGTGGAAAGGACGAAACACCGCTCTATGACTGTCCCATGTGGTTTTAGAGCTAGAAATAGCAAGTT

Supplemental Table 3: Primers used for this study.

Species	Primer name	Sequence (5' to 3')	Purpose
human	*ATXN3*	TCCACGAGAAACAAGAAGGCT (Forward)	RT-qPCR
		TCCTCTCCTCCTCATCCAGC (Reverse)	
human	*CD274*	CCTGCAGGGCATTCCAGAAA (Forward)	
		CCTGCAGGGCATTCCAGAAA (Reverse)	
human	*ACTB*	CGCCGCCAGCTCACC (Forward)	
		AATCCTTCTGACCCATGCCC (Reverse)	
mouse	*Atxn3*	AAGTCGCCAGGAAATCGACA (Forward)	
		GCTGCTGCTGTTGCTTTTCAA (Reverse)	
mouse	*Cd274*	CAGCAACTTCAGGGGGAGAG (Forward),	
		TTTGCGGTATGGGGCATTGA (Reverse)	
mouse	*Actb*	TATAAAACCCGGCGGCGCA (Forward)	
		TCATCCATGGCGAACTGGTG (Reverse)	
	GeCKO-NGS-2	AATGATACGGCGACCACCGAGATCTACACT CTTTCCCTACACGACGCTCTTCCGAT (Forward)	CRISPR NGS
	GeCKO-NGS-3	AATGATACGGCGACCACCGAGATCTACACTC TTTCCCTACACGACGCTCTTCCGATCTGATGC ACATCTGCTTTATATATCTTGTGGAAAGGACG AAACACC (Forward)	
	GeCKO-NGS-KO-1	CAAGCAGAAGACGGCATACGAGATTCGCCTT GGTGACTGGAGTTCAGACGTGTGCTCTTCCGA TCTCCGACTCGGTGCCACTTTTTCAA (Reverse)	
	GeCKO-NGS-KO-2	CAAGCAGAAGACGGCATACGAGATATAGCGT CGTGACTGGAGTTCAGACGTGTGCTCTTCCGA TCTCCGACTCGGTGCCACTTTTTCAA (Reverse)	
	GeCKO-NGS-KO-3	CAAGCAGAAGACGGCATACGAGATGAAGAAG TGTGACTGGAGTTCAGACGTGTGCTCTTCCGAT CTCCGACTCGGTGCCACTTTTTCAA (Reverse)	
	GeCKO-NGS-KO-4	CAAGCAGAAGACGGCATACGAGATATTCTAGG GTGACTGGAGTTCAGACGTGTGCTCTTCCGATC TCCGACTCGGTGCCACTTTTTCAA (Reverse)	
	GeCKO-NGS-KO-5	CAAGCAGAAGACGGCATACGAGATCGTTACCA GTGACTGGAGTTCAGACGTGTGCTCTTCCGATC TCCGACTCGGTGCCACTTTTTCAA (Reverse)	
	GeCKO-NGS-KO-6	CAAGCAGAAGACGGCATACGAGATGTCTGATG GTGACTGGAGTTCAGACGTGTGCTCTTCCGATC TCCGACTCGGTGCCACTTTTTCAA (Reverse)	

	GeCKO-NGS-KO-7	CAAGCAGAAGACGGCATACGAGATTTACGCACGTGACTGGAGTTCAGACGTGTGCTCTTCCGATCTCCGACTCGGTGCCACTTTTTCAA (Reverse)	
	GeCKO-NGS-KO-8	CAAGCAGAAGACGGCATACGAGATTTGAATAGGTGACTGGAGTTCAGACGTGTGCTCTTCCGATCTCCGACTCGGTGCCACTTTTTCAA (Reverse)	
	custom-NGS-KO-9	CAAGCAGAAGACGGCATACGAGATAGGTTATCGTGACTGGAGTTCAGACGTGTGCTCTTCCGATCTCCGACTCGGTGCCACTTTTTCAA (Reverse)	
